# Ash dieback and hydrology affect tree growth patterns under climate change in European floodplain forests

**DOI:** 10.1038/s41598-025-92079-5

**Published:** 2025-03-24

**Authors:** Stefanie Henkel, Ronny Richter, Karl Andraczek, Roger Mundry, Madeleine Dontschev, Rolf A. Engelmann, Timo Hartmann, Christian Hecht, Hans Dieter Kasperidus, Georg Rieland, Mathias Scholz, Carolin Seele-Dilbat, Michael Vieweg, Christian Wirth

**Affiliations:** 1 Systematic Botany and Functional Biodiversity, Institute for Biology, Leipzig University, Johannisallee 21, 04103 Leipzig, Germany; 2 Department of Conservation Biology and Social-Ecological Systems, Helmholtz Centre for Environmental Research (UFZ), Permoserstraße 15, 04318 Leipzig, Germany; 3 German Centre for Integrative Biodiversity Research (iDiv) Halle-Jena-Leipzig, Puschstraße 4, 04103 Leipzig, Germany; 4 Department Biodiversity and People, Helmholtz Centre for Environmental Research (UFZ), Permoserstraße 15, 04318 Leipzig, Germany; 5 Cognitive Ethology Laboratory, German Primate Center, Leibniz Institute for Primate Research, Kellnerweg 4, 37077 Göttingen, Germany; 6 Department for Primate Cognition, Georg-August-Universität Göttingen, Johann-Friedrich-Blumenbach Institute, Kellnerweg 4, 37077 Göttingen, Germany; 7 Leibniz ScienceCampus Primate Cognition, Göttingen, Germany; 8 Department of Plant Ecology, Institute for Ecology, Technische Universität Berlin, Straße des 17. Juni 135, 10623 Berlin, Germany; 9 Department Community Ecology, Helmholtz Centre for Environmental Research (UFZ), Theodor-Lieser- Str. 4, 06120 Halle, Germany; 10 Department of Geobotany and Botanical Garden, Martin Luther University Halle-Wittenberg, Am Kirchtor 1, 06108 Halle, Germany; 11 Anhalt University of Applied Sciences, Nature Conservation and Landscape Planning, 06406 Bernburg, Germany; 12 Agency for Environmental Protection, Nature Conservation Authority, Prager Str. 118-136, 04317 Leipzig, Germany

**Keywords:** Floodplain forest, Tree growth, Ash dieback, Climate change, Hydrology, Forest reorganization, Biodiversity, Forest ecology

## Abstract

Floodplain forests are currently undergoing substantial reorganization processes due to the combined effects of management-induced altered hydrological conditions, climate change and novel invasive pathogens. Nowadays, the ash dieback is one of the most concerning diseases affecting European floodplain forests, causing substantial tree mortality and threatening the loss of the dominant key tree species of the hardwood floodplain forest, *Fraxinus excelsior*. Understanding how the increased light availability caused by pathogen-driven mortality in combination with altered hydrological conditions and climate change affects growth responses in a diverse forest community is of crucial importance for conservation efforts. Thus, we examined growth of the main tree species in response to ash dieback and how it depended on altered hydrological conditions under novel climatic conditions for the lower and upper canopy in the floodplain forest of Leipzig, Germany. Our study period encompassed the consecutive drought years from 2018 to 2020. We found that tree growth responded mostly positively to increased light availability, but only on moist sites, while tree growth largely declined on dry sites, suggesting that water availability is a critical factor for tree species to be able to benefit from increased light availability due to canopy disturbances caused by ash dieback. This hydrological effect was species-specific in the lower canopy but not in the upper canopy. While, in the lower canopy, some species such as the competitive shade-tolerant but flood-intolerant *Acer pseudoplatanus* and *Acer platanoides* benefited from ash dieback on moist sites, others were less affected or suffered disproportionally, indicating that floodplain forests might turn into a novel ecosystem dominated by competitive *Acer* species, which may have detrimental effects on ecosystem functioning. Our results give hints on floodplain forests of the future and have important implications for conservation measures, suggesting that a substantial revitalization of natural hydrological dynamics is important to maintain a tree composition that resembles the existing one and thus sustain their conservation status.

**Supplementary Information:**

The online version contains supplementary material available at 10.1038/s41598-025-92079-5.

## Introduction

Forest ecosystems around the globe are currently undergoing complex reorganization processes due to increasing natural and anthropogenic disturbances, which are further amplified by continuing climate change^1,2^. Disturbances and novel stressors, such as invasive pathogens, wildfires, windthrows, droughts and heat waves, are important drivers of forest ecosystem dynamics, often leading to substantial tree mortality and abrupt changes in growing conditions^1,3–5^. The initial years following a disturbance, the reorganization phase, is crucial, as early colonizers and the dynamics of advanced regeneration in the understory often shape the forest’s structure and composition for decades and centuries to come^1,6,7^. Under climate change and anthropogenic influence, these reorganization processes fundamentally deviate from usual succession dynamics as disturbances nowadays coincide with significantly altered conditions, potentially catalyzing the system’s shift into a new regime and hampering the forests’ provision of ecosystem services^2,8^. Floodplain forests are a prime example of ecosystems currently undergoing significant reorganization processes due to multiple coinciding compound effects of global change. They are also one of the most dynamic, productive and diverse, yet one of the most endangered ecosystems in the world^9–13^. Understanding these novel reorganization processes and post-disturbance growth responses under climate change is of crucial importance for conservation efforts which is why we examine the effect of a novel pathogen under climate change on the growth dynamics of a species-rich and protected floodplain forest.

The stressors floodplain forests currently experience are manyfold. First, extensive hydraulic engineering measures of the past, such as river straightening and dike constructions, led to the absence of floods and sinking groundwater levels, causing floodplains across Central Europe to dry up on a large scale^14–16^. Drainage, coupled with altered silvicultural practices shifting away from coppice-with-standards management, allowed untypical floodplain forest species such as the flood-intolerant but rather shade-tolerant sycamore maple (*Acer pseudoplatanus* L.) to thrive, while keystone species like the light-demanding pedunculate oak (*Quercus robur* L.) are quickly vanishing^17,18^. Second, climate change is associated with increases in the frequency, duration, and/or intensity of drought and heat stress, which could fundamentally alter the composition and structure of these forests^3^, IPCC^19^. From 2018 to 2020, Central Europe was faced with the hottest and driest consecutive years in 250 years of climate records, showing an unprecedented level of intensity^20,21^ which is likely to occur more often as climate change exacerbates^22^. Normally, most floodplain species are adapted to surviving dry summer periods, by either accessing groundwater with their roots or by restricting their water consumption^15^. However, several studies have found temperate floodplain forests to be susceptible to drought-induced stress on tree growth^23–25^, and prolonged or consecutive droughts appear to bring floodplain forests closer to a tipping point^21,24^. Third, invasive pathogens are causing a high degree of tree mortality and changing forest structure, with climate change driving these processes^26^. Due to warmer climatic conditions, winter mortality in biotic agents of forest pests is likely to be reduced, leading to an increase in outbreaks^27^. After the Dutch elm disease spread widely in the last century and has eliminated the majority of elm trees –a typical floodplain forest species – from the overstory^17,28^, the most concerning disease affecting these systems today is the ash dieback, threatening the loss of yet another key tree species of the hardwood floodplain forest. Ash dieback is caused by the fungus *Hymenoscyphus fraxineus* ^29^ and the associated secondary fruit form *Chalara fraxinea*^30^, resulting in severe defoliation and eventual tree mortality^31–33^. Many European floodplain forests have a high percentage of ash in the canopy cover^31,33^, and the imminent collapse of ash populations has far-reaching consequences for biodiversity and the functionality or conservation status of the hardwood floodplain forest^12,34^. Together, these stressors impose critical threats to forest ecosystem functioning.

Tree growth responses to canopy disturbances are complex and depend on various factors, including species characteristics, environmental conditions, and competition^35^. When a canopy gap forms, light availability increases, but it also leads to significant changes in other abiotic factors such as temperature, soil moisture, nutrient availability, and below-ground competition^36,37^. Residual trees have been reported to show increased diameter growth to canopy disturbances, although this response may be delayed due to the rapid change in growth conditions, which can sometimes even cause a temporary decline^35,38–41^. In general, smaller overstory trees may benefit more from increased light and resource availability, showing stronger growth responses than larger trees^35,38,40–42^, whose growth may be further constrained by their age and size^35,43^. Some larger trees might also respond by rapidly filling the newly available space through crown expansion^35,40,44^. This study aims to better understand how trees in different canopy layers react to disturbances caused by ash dieback in these compositionally and structurally complex forest ecosystems.

Species-specific growth responses to canopy disturbances and water availability can be linked to species-specific ecological attributes, such as shade or drought tolerance, and specific demands for water and light. Most studies examining growth responses related to shade tolerance focus on individuals in the juvenile stage^45,46^, whereas little research was done on mature individuals, and results here are ambiguous. Shade-intolerant species were found to react stronger to canopy disturbance^47^, whereas other studies found shade-tolerant species, especially the smaller individuals, to respond strongly to canopy openings^38^. Drought resistance might affect growth responses under altered hydrological conditions, with drought resistant species coping better on dry sites as compared to drought sensitive species^48^.

In this study, we aimed at investigating one key aspect of early reorganization processes in response to a natural disturbance under novel climatic conditions in a diverse European floodplain forest system. Specifically, we examined growth responses of the main tree species to canopy disturbances and thus increased light availability caused by a fungal pathogen, the ash dieback, in dependence on altered hydrological conditions in the floodplain forest of Leipzig, Saxony, Germany. Due to their high tree species richness^49^, floodplain forests are ideal for comparative studies of tree species reactions to disturbances depending on water availability as they are among the few ecosystems with coexisting mature trees showing a wide range of water-use strategies^24,50^. We hypothesized that:

1. All species in general show positive growth responses to increased light availability due to ash dieback,
2. The effect of ash dieback on growth responses is dependent on hydrological conditions, with more pronounced positive effects on moist sites as compared to dry sites,
3. Trees in different canopy strata react in a different way: (a) trees in the lower canopy particularly benefit from canopy disturbance as the relative increase in access to light is higher than in upper tree layers, (b) trees in the upper canopy show weak responses to ash dieback as they are less likely to be limited by competition for light, and
4. Due to expected differences in shade tolerance and water demands, growth responses to changing light and hydrological conditions will differ between species.

## Materials and methods

### Study site & design

In this study, we used data collected in the northwestern part of the floodplain forest in Leipzig, Saxony, Germany, within the scope of the project ‘Lebendige Luppe’^51^. The Leipzig floodplain forest is one of the largest floodplain forests in Central Europe^10,52^ and highly protected due to its relevance as biodiversity hotspot^14,21,53^. Up to 1870, the Leipzig floodplain forest was managed by coppice-with-standards silvicultural approaches. Since then, the forest has remained largely undisturbed and has been formally protected as a nature reserve since the 1990s. The prevailing climate is continental with an average annual temperature of 9.7 °C and a mean annual precipitation of 520 mm (1980–2020; DWD Climate Data Center [CDC], Station Leipzig/Halle, ID 2932; for a climatic diagram and SPEI index see^54^). It is situated along the rivers Weiße Elster, Pleiße, Luppe and Parthe and has been shaped by human interventions over the last centuries^55^. Extensive hydraulic engineering schemes such as dike construction and river regulations altered the hydrological regime. Not only did it prevent natural flooding for over 70 years, but the increasing deepening of the river also led to generally lower groundwater levels in the surrounding floodplain^10,56^. The dominant soil type can be characterized as a loamy Vega that developed on a 2–4 m thick layer of clay deposited after upstream soil erosion since 8000 b.c. on quarternary alluvials sediments dominated by gravel and sand^10,57^. The consecutive drought years 2018, 2019 and 2020 have created a large deficit in the plant-available water in the soils of Saxony, which affected the ecosystem of the floodplain forest of Leipzig particularly severely and accelerated the spread of tree diseases^21,24^, as the regional water balance was already disrupted by flood prevention measures and a lowered groundwater tables^34,56^.

The study design of the ‘Lebendige Luppe’ project encompasses 60 permanent observation plots each 0.25 ha in size which cover a gradient in topographic distances to the groundwater level^58^. We used a stratified sampling design with three strata of distance to groundwater: dry (> 2 m), intermediate (1–2 m) and moist (≤ 1 m) plots to represent the entire hydrological gradient of the floodplain, with 20 plots per stratum. During the course of the project, higher resolution hydrological data loggers were installed, allowing the calculation of tree-based groundwater to surface distances using a coupled hydrological model^59,60^, see 3.2. The study site has missed regular flooding since 1954 due to flood control measures (dikes, river-straightening etc.), except for winter 2011 and summer 2013, when the area of Leipzig experienced extreme flood events. All plots are located in areas designated as FFH habitat type hardwood floodplain forest (LRT 91F0*) or as starmason-oak-hornbeam-forest on dry sites (LRT 9160, here degradation form of the hardwood floodplain forest due to the absence of flooding^61^) or other forest stands with an age of ≥ 80 years. *F. excelsior*, *Q. robur* and *Ulmus spec.* are the characteristic hardwood floodplain forest species and crucial for its conservation status as FFH habitat type, whereas *Acer* species, above all *A. pseudoplatanus*, are untypical for this forest type and were historically rare or absent^21,62,63^. *F. excelsior* is by far the most dominant species, thus making its loss through ash dieback detrimental for forest composition and ecosystem functioning.

### Hydrological data

Hydrological conditions were continuously measured on 34 of the 60 plots via groundwater measuring stations reaching 4 m into the ground^58^. The plots for the installation of the groundwater monitoring stations were selected to include a gradient of the surface to groundwater distance. The groundwater conditions are mostly confined or at least semiconfined by a 2.5–3.5 m thick low permeable alluvial clay layer^57^. The measuring points are groundwater observation wells consisting of HDPE pipes (DN 32/25 mm) with a filter section at the level of the aquifer that was sealed with Bentonite granules. Pressure loggers were installed in the wells to measure the groundwater level (groundwater potential) and temperature in high temporal resolution (TD-Diver Schlumberger Water Services, half-hourly measuring interval). The water levels were converted to absolute heights above sea level using precise D-GPS measurements. Data from > 50 groundwater observation wells were used for calibrating a coupled groundwater surface water model with daily means for a 30 m grid of the research area. We calculated tree-based distances to groundwater by subtracting the modeled groundwater surface (based on a coupled groundwater surface water model)^59^ from the ground level elevation for each individual tree.

### Tree data

Two forest inventories were carried out which monitored all living tree and shrub individuals with a diameter at breast height (DBH) of ≥ 5 cm on the whole plot area (see Table S1 Supplementary Material)^64,65^. The first inventory took place in two time periods: for the initial 31 plots in the winter of 2013/14, and after the plot network was extended, the remaining 29 plots were inventoried in the winter of 2016/17. The second inventory took place in the winter of 2020/21 for all plots. Thus, our study period encompassed the severe consecutive drought years from 2018 to 2020, allowing us to study indirect effects of climate change on growth responses of the main tree species.

The horizontal and vertical position of the trees, as well as the measurement of tree height and crown base height, were recorded using optical tachymetric surveying. The precise location of the measuring device was determined with the aid of satellite navigation measurements in an official coordinate system, achieving an accuracy of at least 2 cm with RTK (Real Time Kinematic) correction data. With the help of the exact tree positions, we could determine the distance to groundwater for each individual tree via the ground level elevation (see 2.2). Furthermore, for each individual, the DBH was measured, the tree species was determined and its social class (1–6 according to Kraft’s classification^66^; 1 = predominant, 2 = dominant, 3 = co-dominant, 4 = dominated, 5 = sheltered, 6 = remnants), the degree of canopy cover (fully covered, partially covered, uncovered), the vitality status (vital, normal, suffering) and the degree (1–3) and type of damage (stem damage, crown breakage, dry parts in the crown, visible stem rot, fruit bodies of tree fungi at the trunk, other fungi or damage) was assessed. All trees were tagged with a numbered plaque for better identification in the field^65^.

### Data on ash dieback

In order to monitor the extent and development of ash dieback in the Leipzig floodplain forests, annual evaluations of all ash trees (*n* = 1075) on the 60 plots are carried out since 2016^67,68^. We used a standardized 6-step classification method (from damage class 0 = healthy to damage class 5 = dead or dying) to document the symptoms of the infestation^58,67,69–71^. For the following analyses, we used the data of the ash-dieback assessment of 2017 and 2020 and only included predominant, dominant and co-dominant ash trees.

Ash dieback was first observed in Poland in 1992 and has since then spread to 22 European countries^72^; in the Leipzig floodplain forest, it was first documented around 2009^21,73^. The symptoms of the ash dieback can be first observed in young ash stands through discolouration of the shoots, as well as through wilting, leaf spots, and bark necrosis^69^. For mature trees, relevant characteristics of the ash dieback are loss of leaves, dead twigs or branches, and in some cases the formation of a secondary crown^69^. The terminal stages of the disease lead to excessive foliage loss and ultimately to mortality^69,71^, creating gaps in the forest canopy. By 2020, 71% of all assessed ashes in the floodplain forest of Leipzig showed intermediate to strong damage symptoms, while not a single assessed individual was without symptoms^21,73,74^.

### Data analysis

#### Data Preparation

Prior to data analysis, we checked the data for plausibility and removed cases with inconsistencies regarding the DBH measurements, such as missing measurements or deviations from the standardized measuring height of 130 cm by at least 10 cm (44 out of 8624 total individuals). For the final analysis, we only considered the main tree species with the highest abundances in the Leipzig floodplain forest, namely *A. campestre*,* A. platanoides*,* A. pseudoplatanus*,* C. betulus*,* Q. robur*,* T. cordata* and *Ulmus spec.* (including *U. minor*,* U. glabra* and *U. x hollandica*), and only included tree individuals that were present during both the first and the second inventory to be able to calculate relative growth rates (for a flowchart summarizing the data preparation workflow see Fig. S1 Supplementary Material).

To assess the effect of ash dieback and hydrological conditions on growth responses for different canopy layers (see hypothesis 3), we categorized trees into upper and lower canopy individuals according to their position in the stand’s social structure. The position of a tree in the stand and, thus, its growth potential is best described by its social class^66^. Therefore, we combined Kraft’s classes 1–3 (predominant, dominant, co-dominant) to ‘upper canopy’ (UC) and classes 4–5 (dominated and sheltered) to ‘lower canopy’ (LC).

To assess the damage degree of individual trees, we summed up the degree of all eight damage types per tree individual and inventory, and calculated the difference between damage degrees of the second and first inventory (*Δ damage degree*) to get an index of change in tree individual health conditions.

As a proxy for competition, we calculated plot density as the total basal area (*BA*) of all trees per plot (including ash trees, shrubs and less abundant species).

In order to approximate the magnitude of the change in light availability due to ash dieback, we calculated the effective basal area change for all ash trees in the UC (dominant, pre-dominant and co-dominant individuals) per plot. To this end, we used the range of defoliation for each damage category in the 6-step classification according to Peters et al.^70,71^ to calculate the mean defoliation difference from 2020 to 2017 as an indicator for the change in light conditions. We multiplied the defoliation difference for each individual tree with the ash basal area of the first inventory to calculate the effective ash basal area change for each tree and summed it up per plot. We assume that the higher the effective ash basal area change (hereafter ash dieback intensity), the more light became available on the plots from the first to the second inventory.

As a proxy for water availability, we used the median distance to groundwater for the summer months from beginning of May until the end of October over the years from 2014 to 2020 for each individual tree. We excluded the year 2013 as in this year there was a severe flooding in Leipzig which biased the mean values for the following years.

We calculated the relative growth rate of each individual tree as

$$\:RGR=\frac{\left(\frac{\left(BA2-BA1\right)}{\varDelta\:T}\right)}{BA1}$$

with BA1 being the basal area in the first inventory, BA2 the basal area in the second inventory, and ΔT the time interval between the two inventories.

#### Statistical analysis

We used Linear Mixed Models (LMMs)^75^ to estimate the effect of canopy disturbance due to ash dieback and hydrological conditions on growth responses of the main tree species in the Leipzig floodplain forest. We furthermore expected different growth reactions in the LC and in the UC. To avoid model stability issues and hardly interpretable results due to an unbalanced dataset in terms of species and canopy layer distribution, we ran two separate models for LC and UC trees, respectively, and excluded *Q. robur* in the LC model and the small-statured *A. campestre* and *Ulmus spec.* in the UC model as there are hardly any oaks in the understorey and hardly any individuals of *A. campestre* and *Ulmus spec*. in the overstorey (see Table 1). Model structure was otherwise identical for both models. In both models, we used the relative growth rate (RGR) as response variable and included species, ash dieback and distance to groundwater as fixed effects (test predictors). We also included all interactions up to order three between all test predictors as we expected species-specific growth responses in dependence on the different environmental factors. To control for the effect of resource competition on growth responses, we included plot density as a fixed effect. To control for individual tree growth conditions, we also included Δ damage degree and the vitality state of each tree in the second inventory as fixed effects. To control for the different timespans between the first and second inventory, we furthermore included the time difference in years between the first and second inventory (ΔT) as fixed effect. We included plot ID as random intercepts effect to control for plot specific effects. Furthermore, we had 129 trees that had more than one stem below breast height of 130 cm (“Zwiesel”). In order to control for non-independent growth responses of these bifurcated trees, we included tree ID as an additional random intercepts effect. Tree ID is defined as the tree including all of its forks. To avoid the model being overconfident with regard to the precision of fixed effects estimates and to keep type I error rate at the nominal level of 5%, we included all theoretically identifiable random slopes^76,77^. More precisely, we included random slopes of species, distance to groundwater, Δ damage degree and vitality within plot ID (species and vitality were manually dummy coded and then centered before entering them into the random effects part). We also included parameters for the correlations among random intercepts and slopes.

**Table 1:** Numbers of individuals per species that were present in both the first and second inventory for the lower and upper canopy, respectively.

Species	Lower canopy	Upper canopy
*Acer campestre*	368	21
*Acer platanoides*	392	43
*Acer pseudoplatanus*	786	270
*Carpinus betulus*	475	74
*Quercus robur*	26	311
*Tilia cordata*	494	153
*Ulmus spec.*	1070	26
Total	3611	898

As an overall test of the effects of the test predictors and their interactions, we conducted a full-null model comparison, aiming at avoiding cryptic multiple testing^78^, whereby the null model lacked the test predictors and their interactions in the fixed effects part but was otherwise identical to the full model. This comparison was based on a likelihood ratio test (LRT)^79^. We tested the effect of individual fixed effects by means of the Satterthwaite approximation^80^ using the function lmer of the package lmerTest (version 3.1.3)^81^ and a model fitted with restricted maximum likelihood.

Prior to fitting the model, we inspected all quantitative predictors and the response for whether their distributions were roughly symmetrical. As a consequence, we log-transformed RGR (after adding 0.01). We only used values of RGR > 0. We then z-transformed ash dieback, plot density, distance to groundwater, Δ damage degree and ΔT to achieve an easier interpretable model^82^ and ease model convergence.

After fitting the model, we checked whether the assumptions of normally distributed and homogeneous residuals were fulfilled by visual inspection of a QQ-plot of residuals^83^ and residuals plotted against fitted values^84^. These indicated no deviations from these assumptions. Collinearity, determined for a model lacking the interactions, appeared to be no issue (maximum Variance Inflation Factor: 1.20)^84^. We checked for model stability by excluding the levels of the grouping factor plot ID one at a time from the data and comparing the estimates derived with those obtained for the model based on all data^85^, which indicated no influential cases to exist.

We conducted post-hoc pairwise comparisons to compare growth responses to ash dieback between high and low distances to groundwater (i.e. on dry and moist sites, respectively) for each species as well as between all species for dry and moist sites, respectively, separately for the lower and upper canopy. To do so, we used the 1000 fitted values obtained through parametric bootstrapping to determine the slopes against ash dieback for the 25% and 75% quantiles of distance to groundwater, respectively, and calculated the difference between the slopes per bootstrap. We then determined the proportion of slope differences smaller than or equal to zero and the proportion of slope differences larger than or equal to zero, took the smaller of the two proportions and multiplied it by two to obtain two-tailed p-values.

To test whether growth rates generally differ between the UC and LC for the different species, we additionally ran a LMM including all species. We used RGR (log-transformed) as response variable and canopy layer and species as fixed effects. We also included the interaction between canopy layer and species and plot ID as random intercepts effects to control for plot specific effects. We performed post-hoc pairwise comparisons to test for differences between UC and LC per species using the function emmeans of the package emmeans (version 1.10.6). Note that we could not include random slopes into this model, and hence the resulting p-values for the pairwise comparisons will be anti-conservative (i.e. too small).

We fitted the models in R (version 4.3.1)^86^ using the function lmer of the package lme4 (version 1.1.34)^87^ and lmerTest (version 3.1.3)^81^. We determined Variance Inflation Factors using the function vif of the package car (version 3.1.2)^88^. We assessed model stability using a function written by RM. We derived confidence intervals using the function bootMer of package lme4, using 1,000 parametric bootstraps and bootstrapping over the random effects, too. We calculated marginal R^2^-values using the function r.squaredGLMM of the package MuMIn (version 1.47.5)^89^. We set significance at *p* < 0.05 and trends at 0.05 ≤ *p* < 0.1. Computations for this work were done in part using resources of the iDiv High-Performance Computing (HPC) cluster in Leipzig.

The sample size for the LC model encompassed 3585 trees, with 3399 tree IDs nested in 59 plot IDs. The total number of estimated effects for this model was 86, with 41.7 data points per estimated effect. The sample size for the UC model encompassed 851 trees, with 843 tree IDs nested in 59 plot IDs. The total number of estimated effects for this model was 72, with 11.8 data points per estimated effect. Marginal R^2^-values were 0.196 for both the UC model and the LC model. The sample size for the model testing for differences between canopy layers encompassed 4726 trees and its marginal R^2^-value was 0.137. According to the suggestion of a reviewer, we determined R^2^ as a measure of model fit with respect to ash dieback and distance to ground water separately per species as follows: we first determined fitted values with respect to species, ash dieback, and distance to ground water for both the LC and the UC model. We then determined Pearson’s correlation coefficient between these fitted values and the response, separately for each species and finally squared them to obtain an R^2^-like measure of how well the model with respect to species, ash dieback and distance to ground water fitted the observed response.

## Results

### Characterization of the ecosystem

The dominant species in the study area, both in the first and the second inventory of the tree layer (first inventory: *N* = 7139, second inventory: *N* = 7610), with decreasing relative dominance, were common ash (*Fraxinus excelsior* L.), pedunculate oak (*Quercus robur* L.), sycamore maple (*Acer pseudoplatanus* L.), small-leaved lime (*Tilia cordata* Mill.), hornbeam (*Carpinus betulus* L.), elm (*Ulmus spec*., including the three elm species field, flutter and mountain elm - *Ulmus minor* Mill., *U. laevis* Pall., *U. glabra* Huds. – and their hybrids, e.g. U. x *hollandica* Mill.), field maple (*Acer campestre* L.), and Norway maple (*Acer platanoides* L.; see Fig. S2 Supplementary Material). *F. excelsior*, comprising about 40% of the canopy, is the by far most dominant species.

**Fig. 1:**
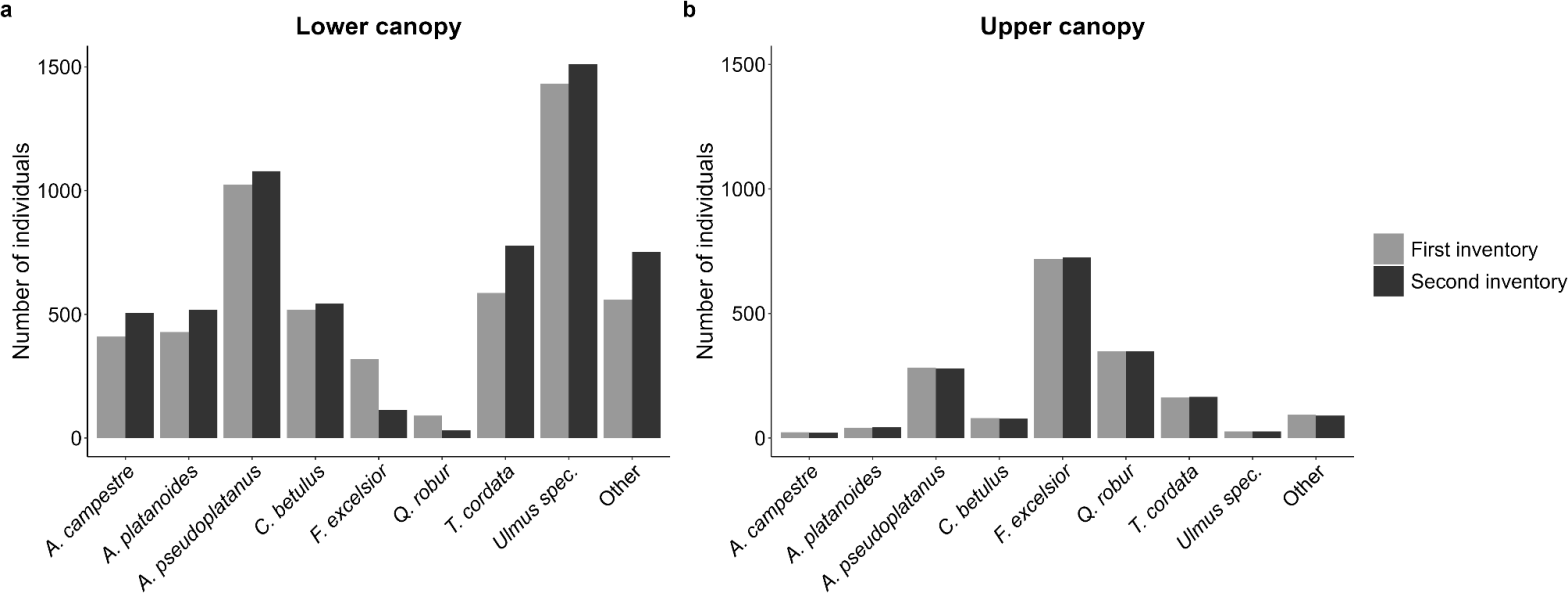
Total number of individuals (DBH ≥ 5 cm) of the main tree species in the Leipzig floodplain forest in the first and second inventory for ** (a)** the lower canopy and **(b)** the upper canopy. Sample sizes for the upper canopy: first inventory: *N* = 1775, second inventory: *N* = 1777. Sample sizes for the lower canopy: first inventory: *N* = 5365, second inventory: *N* = 5832. Plots: *N* = 60 (0.25 ha each).

**Fig. 2:**
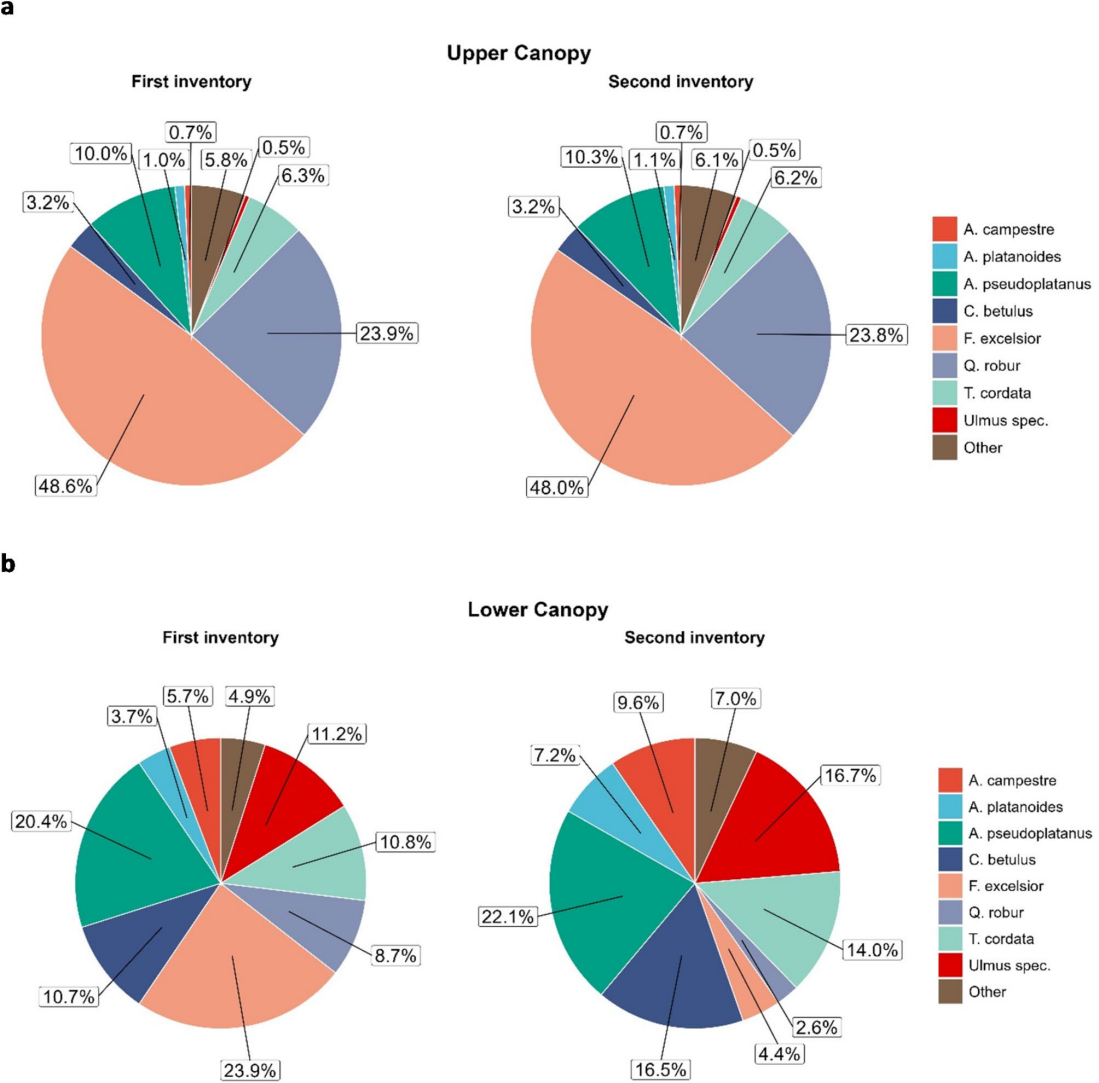
Species composition (relative dominance) based on basal area in the Leipzig floodplain forest in the first and second inventory for **(a)** the upper and **(b)** the lower canopy. Sample sizes for the upper canopy: first inventory: *N* = 1775, second inventory: *N* = 1777. Sample sizes for the lower canopy: First inventory: *N* = 5365, second inventory: *N* = 5832.

**Fig. 3:**
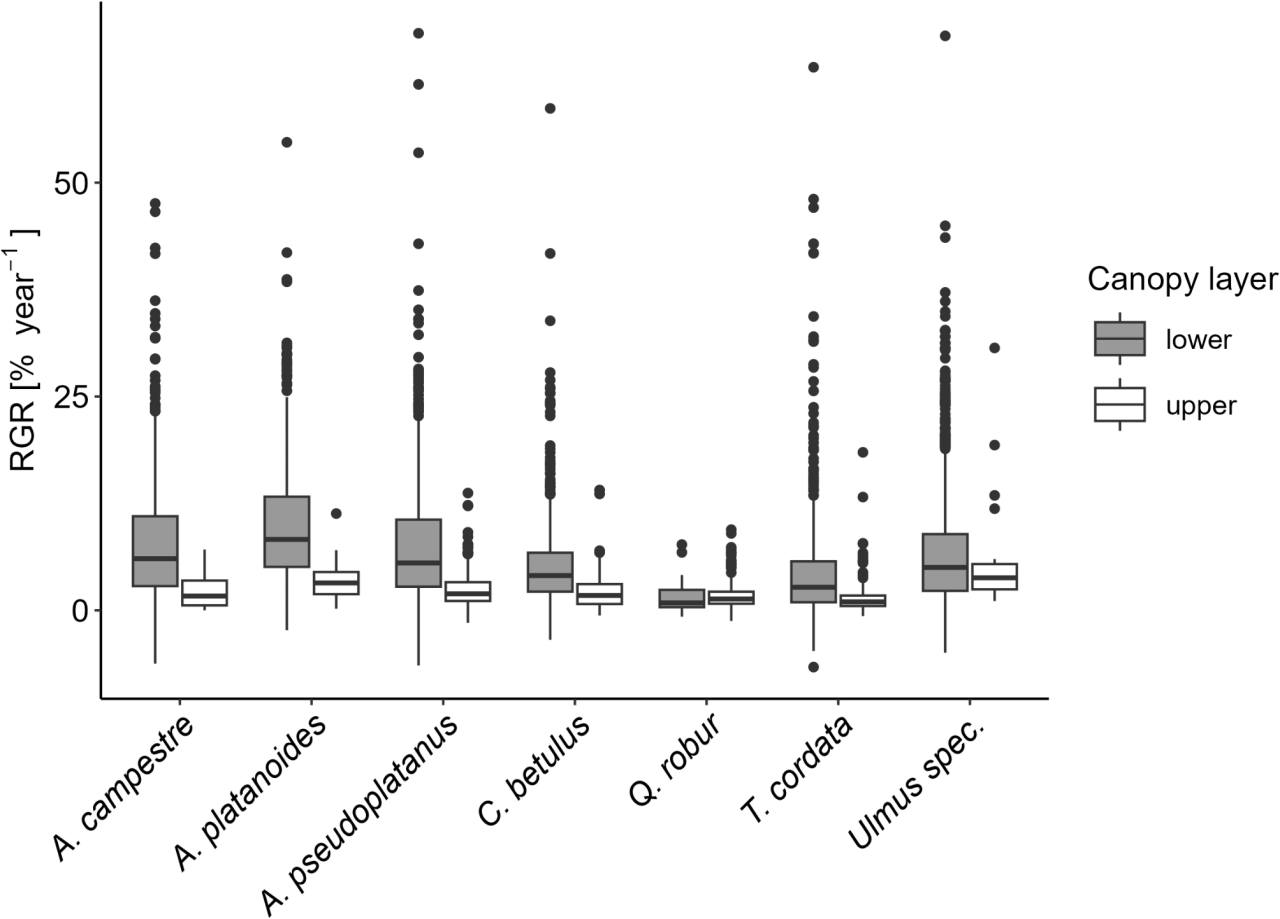
Relative growth rate (RGR) per species and canopy layer. Lines within the boxes indicate the median. Includes all RGR (*N* = 4726).

When taking the different tree layers into account, it became evident that the number of individuals and the species composition in the upper canopy were relatively stable over the course of the study period (Figs. 1b and 2a). In the lower canopy, however, all species increased in numbers and relative dominance from the first to the second inventory except *F. excelsior* and *Q. robur* whose numbers and relative dominance drastically decreased (Figs. 1a and 2b). In the lower canopy, *Acer* species together made up 29.8% of the species composition in the first inventory and 38.9% in the second inventory. For presenting descriptive data characterizing stand structure dynamics we used diameter classes. Distributions of annual basal area increments per ha per DBH class showed that while increments in *F. excelsior* and *Q. robur* were primarily due to an increase in basal area in high diameter classes (from DBH = 40 cm), increments in the three *Acer* species – particularly *A. pseudoplatanus* – were basically based in the advanced regeneration (DBH = 5 to 40 cm), and were 3.5 times higher than those of *F. excelsior* and *Q. robur* combined (0.11 vs. 0.03 m² ha^− 1^ yr^− 1^), already accounting for 24% of the total growth (Fig. S6 Supplementary Material). Recruitment, here defined as the number of individuals (or gain in basal area, respectively) that grew into the DBH class > 5 cm, was highest for *Ulmus* and *A. pseudoplatanus* and lowest for *F. excelsior*, while there was no recruitment at all for *Q. robur* (Fig. S7 Supplementary Material). Median annual relative growth rates (RGR) were highest for *A. platanoides*, followed by *A. campestre*, *Ulmus spec*. and *A. pseudoplatanus*, and lowest for *Q. robur* (see Fig. 3; Table 2). We found a significant interaction between canopy layer and species (F_[6,4678.6]_ = 5.8423, *p* < 0.001, see Table S2). RGRs were significantly higher in the lower as compared to the upper canopy for all species except *Q. robur* and *Ulmus spec.* (Fig. 3, Table S3). Overall, distance to groundwater was not significantly correlated with ash dieback (Pearson correlation: *r*= – 0.192, t= – 1.476, df = 57, *p* = 0.146, Fig. S9c Supplementary Material).

**Table 2:** Median and interquartile range of annual relative growth rate (RGR) per species. *N* = 4726.

Species	Median RGR	0.25% quantile	0.75% quantile
*A. campestre*	0.056	0.026	0.104
*A. platanoides*	0.076	0.044	0.124
*A. pseudoplatanus*	0.042	0.019	0.083
*C. betulus*	0.036	0.018	0.062
*Q. robur*	0.013	0.007	0.022
*T. cordata*	0.020	0.008	0.047
*Ulmus spec.*	0.050	0.023	0.089

### Upper canopy model

**Fig. 4:**
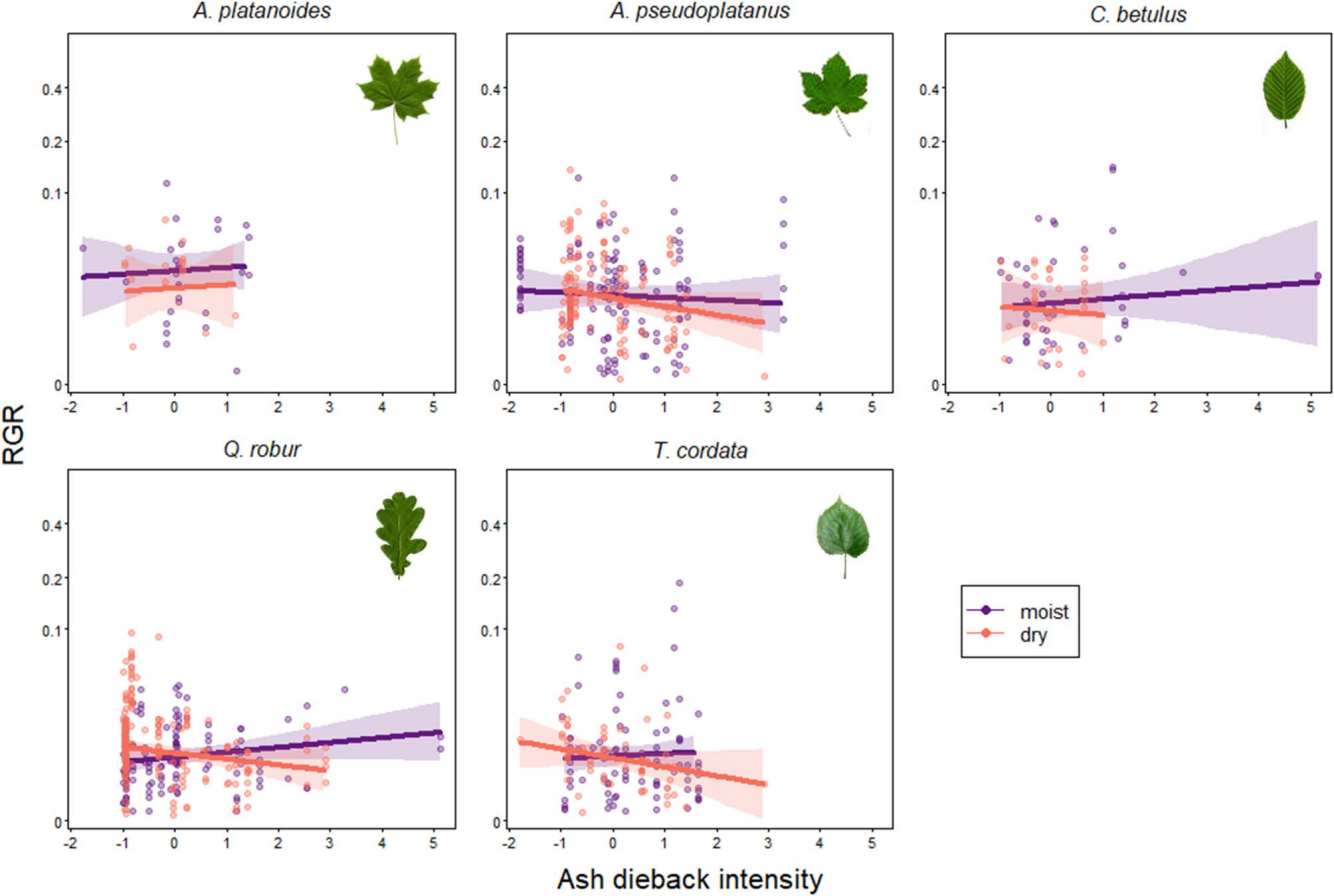
Growth responses to ash dieback (as a proxy for light availability) and how they depend on hydrological conditions for the main tree species in the upper canopy of the Leipzig floodplain forest. Relative growth rates (RGR) in relation to ash dieback intensity are shown for moist (25% quantile of distance to groundwater) and dry sites (75% quantile distance to groundwater). The interaction between species, ash dieback and distance to groundwater on RGR was not significant (see Table S4). Solid lines and shaded areas depict the fitted model and its 95% confidence limits for all other predictors being centered to a mean of zero. *N* = 851 individuals. R^2^-values per species: *A. platanoides*: 0.006, *A. pseudoplatanus*: 0.038, *C. betulus*: 0.031, *Q.robur*: 0.173, *T. cordata*: 0.032.

Overall, the set of predictor variables had a significant effect on the relative growth rate for trees in the upper canopy (UC; full null model comparison: χ^2^ = 48.200, df = 19, *p* < 0.001). However, the 3-way interaction between species, ash dieback and distance to groundwater was not significant for the UC (F_[4,55.11]_ = 0.194, *p* = 0.940, Fig. 4, Table S4 Supplementary Material), suggesting no species-specific growth reactions to ash dieback in dependence on hydrological conditions. In order to obtain interpretable p-values for the lower order interactions, a reduced UC model not comprising the 3-way interaction was fitted. The reduced model revealed a significant effect of the interaction between ash dieback and distance to groundwater (F_[1,27.96]_ = 7.929, *p* = 0.009) but not for the interactions between species and ash dieback (F_[4,40.76]_ = 0.397, *p* = 0.810) nor for the interaction between species and distance to groundwater (F_[4,38.40]_ = 1.217, *p* = 0.320, Table S5 Supplementary Material). In order to obtain an interpretable p-value for species, a second reduced model not comprising the non-significant interactions was fitted. This final model revealed a significant effect of species (F_[4,31.78]_ = 13.095, *p* < 0.001) and of the interaction between ash dieback and distance to groundwater (F_[1,37.66]_ = 10.497, *p* = 0.003, Table S6 Supplementary Material), suggesting that species generally differed in relative growth rates and that growth responses to ash dieback generally varied depending on hydrological conditions.

As expected from the non-significant three-way interaction between species, ash dieback and distance to groundwater, post-hoc pairwise comparisons of growth responses to ash dieback between moist and dry sites and between all species on moist and dry sites did not reveal significance (Table S10 and Table S11 Supplementary Material).

Furthermore, plot density (estimate=-0.086, F_[1,33.07]_ = 7.727, *p* = 0.009, Fig. S10a, Table S6 Supplementary Material) and Δdamage degree (estimate=-0.071, F_[1,61.83]_ = 15.547, *p* < 0.001, Fig. S10b, Table S6 Supplementary Material) had a clear negative effect on relative growth rates in the UC, indicating that growth responses were lower the higher the initial stand density was and the more severely trees got damaged over the years. In contrast, growth responses increased with higher tree vitality in the UC (estimate_[vitality.normal]_ = 0.179, F_[2,32.92]_ = 4.526, *p* = 0.018, Fig. S11a, Table S6 Supplementary Material). The time interval between inventories did not have a significant effect in the UC (estimate=-0.004, F_[1,42.10]_ = 0.022, *p* = 0.882, Table S6 Supplementary Material).

### Lower canopy model

**Fig. 5:**
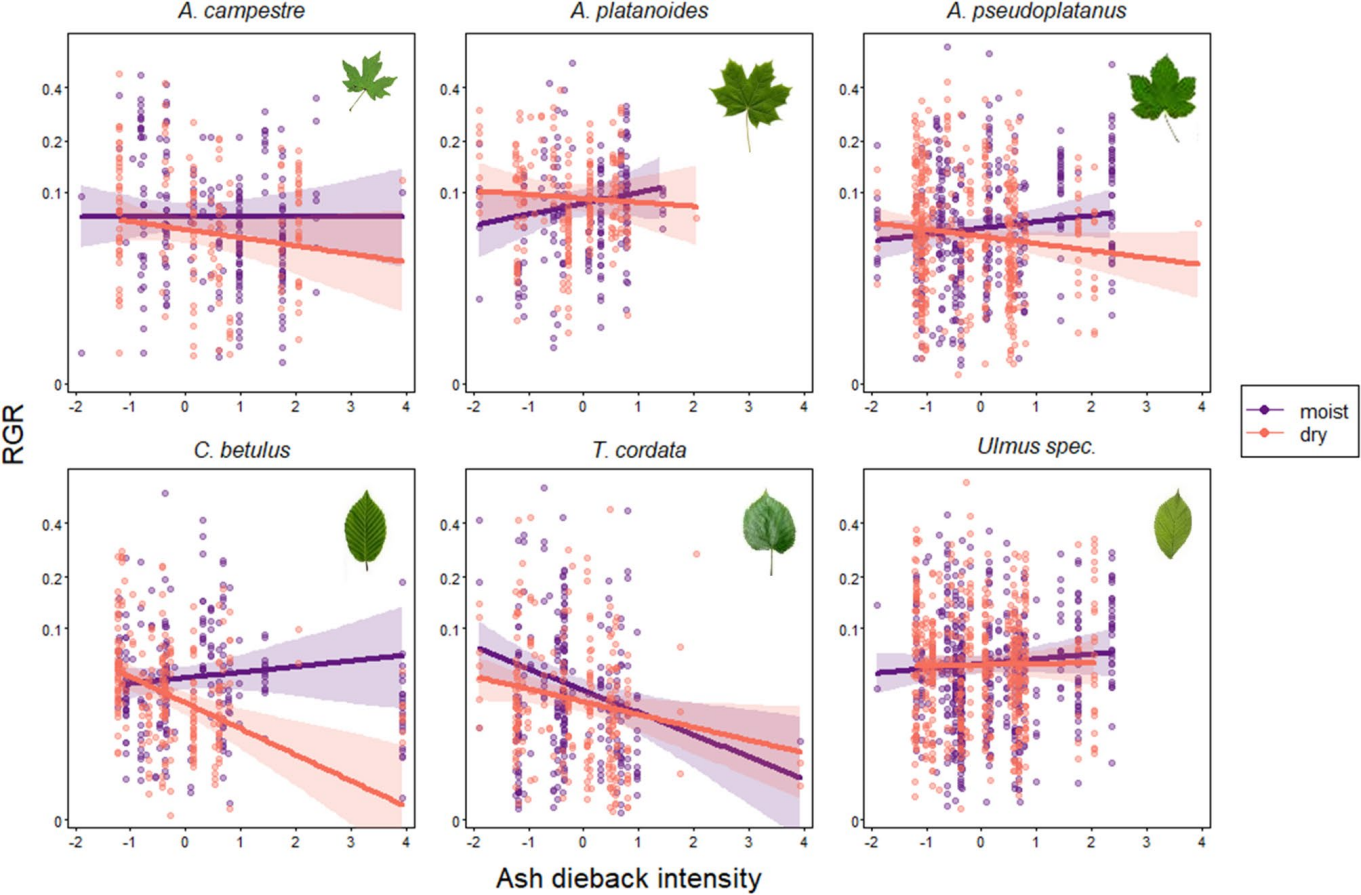
Growth responses to ash dieback (as a proxy for light availability) and how they depend on hydrological conditions for the main tree species in the lower canopy of the Leipzig floodplain forest. Relative growth rates (RGR) in relation to ash dieback intensity are shown for moist (25% quantile of distance to groundwater) and dry sites (75% quantile distance to groundwater). The interaction between species, ash dieback and distance to groundwater on RGR was significant (see Table S7 Supplementary Material). Pairwise post-hoc comparisons between moist and dry sites showed significant differences for *Acer campestre*, *Acer pseudoplatanus* and *Carpinus betulus*. Results for pairwise post-hoc comparisons between species for moist and dry sites, respectively, are shown in Table S10 Supplementary Material. Solid lines and shaded areas depict the fitted model and its 95% confidence limits for all other predictors being centered to a mean of zero. *N* = 3585 individuals. R^2^-values per species: *A. campestre*: 0.001, *A. platanoides*: 0.023, *A. pseudoplatanus*: 0.049, *C. betulus*: 0.097, *T. cordata*: 0.025, *Ulmus spec*.: 0.010.

Overall, the set of predictor variables had a significant effect on the relative growth rate for trees in the lower canopy (LC; full null model comparison: χ^2^ = 99.175, df = 23, *p* < 0.001). More specifically, the 3-way interaction between species, ash dieback and distance to groundwater was significant for the LC (F_[5,108.75]_ = 3.327, *p* = 0.008, Fig. 5, Table S7 Supplementary Material), suggesting species-specific growth responses to canopy disturbance depending on hydrological conditions.

Post-hoc pairwise comparisons revealed significant or highly significant differences, respectively, of growth responses to ash dieback between high and low distances to groundwater for *A. campestre*, *A. pseudoplatanus* and *C. betulus* in the LC, with all species reacting negatively on dry sites and *C. betulus* showing the highest differential response (*A. campestre*: *p* = 0.034, *A. pseudoplatanus*: *p* = 0.006, *C. betulus*: *p* < 0.001; Table S10 Supplementary Material). When comparing species’ growth responses to ash dieback on moist sites, it became evident that *T. cordata* reacted significantly different to all other species, with growth responses decreasing with increasing ash dieback, whereas all other species reacted positively to increasing ash dieback on moist sites (see Table S12 Supplementary Material, Fig. 5). When comparing the growth responses of all species on dry sites, it became clear that the negative growth response to ash dieback in *C. betulus* was significantly stronger as compared to most other species (see Table S12 Supplementary Material, Fig. 5).

It has to be taken into account, though, that the effect of distance to groundwater was based on a continuous variable and comparisons between high and low distances to groundwater are conditional on 25% or 75% percentiles for distance to groundwater, respectively; thus, effects would be more or less pronounced when higher or lower percentiles were chosen. However, 25% and 75% represent a good amount of data which is why we chose these thresholds.

Similar to the UC, plot density (estimate=-0.078, F_[1,55.60]_ = 7.347, *p* = 0.009, Fig. S12a, Table S7 Supplementary Material) and Δdamage degree (estimate=-0.083, F_[1, 55.29]_ = 28.545, *p* < 0.001, Fig. S12b, Table S7 Supplementary Material) had a clear negative effect on relative growth rates in the LC, indicating lower growth responses with higher initial stand density and more severe tree damages over the years. In contrast, growth responses increased with higher tree vitality in the LC (estimate_[vitality.normal]_ = 0.331, F(2,67.38) = 50.593, *p* < 0.001, Fig. S11b, Table S7 Supplementary Material). The time interval between inventories had a significantly negative effect in the LC (estimate=-0.069, F_[1,56.34]_ = 5.100, *p* = 0.028, Table S7 Supplementary Material).

## Discussion

In this study, we investigated growth responses as an important component of the reorganization processes caused by a novel biotic disturbance in combination with novel environmental conditions in a Central European floodplain forest. Our goal was to determine how the main tree species respond to increasing canopy disturbance caused by ash dieback under different hydrological conditions, while disentangling growth reactions in different canopy layers. We found seemingly different growth patterns for the lower and the upper canopy that were dependent on hydrological conditions. Our results indicate that, even shortly after light regime shifts caused by ash dieback, tree species could benefit from increased light availability, albeit only on moist sites, whereas they suffered on dry sites. This effect was species-specific and pronounced in the lower canopy but not in the upper canopy. While, in the lower canopy, some species such as the competitive *A. pseudoplatanus* and *A. platanoides* profited from ash dieback on moist sites, others were less affected or suffered disproportionally, suggesting that species benefitting from ash dieback now will be an even stronger competitive species to others in the future. Our results give hints on the tree species distribution of the floodplain forest of the future and have important implications for conservation measures, suggesting that revitalization of natural hydrological dynamics is important to maintain a tree composition that resembles the existing one.

### General growth responses to Ash dieback and effect of hydrological conditions

Contradicting our first hypothesis, species did not generally profit from increased light availability caused by ash dieback. Growth responses to increasing ash dieback intensity were positive on moist sites (except for *A. campestre* and *T. cordata* in the lower canopy), but negative on dry sites (except for *Ulmus* spp. in the lower canopy). This suggests that water availability is a critical factor for tree species to be able to profit from increased light availability due to canopy disturbances, supporting our second hypothesis that growth responses to ash dieback are dependent on hydrological conditions. Most floodplain forest tree species usually develop a shallow root system as an adaptation to the ample water availability in floodplains, relying more on surface water and top soil moisture^90,91^, with the disadvantage that especially in dry periods there is limited connectivity to the groundwater. Thus, when the groundwater level drops, roots may lose contact to the groundwater and the negative effect of hot summers as experienced during the 2018–2020 consecutive droughts may increase and cause drought stress, usually resulting in reduced growth rates^23,24,92,93^. Under drought conditions, trees may allocate resources differently, e.g. prioritizing root growth over radial growth to enhance their ability to access water^94^. Thus, although canopy disturbance may improve light availability, the remaining trees may only benefit after a significant delay – if at all – because root expansion is necessary first to alleviate the constraints on water supply^95^. Furthermore, it has been shown that released individuals of temperate species are often physiologically stressed before gradually acclimating to new brighter conditions, and that it might take several years for growth release to occur, especially in younger trees^96^. As the time interval between our two inventories was quite short, not enough time might have passed for the trees to adapt to the novel light regime and growth responses might differ if observed over longer periods between canopy release and growth response. It is also possible that under prolonged drought, trees might not benefit at all from increased light availability but rather suffer due to increased drought stress. In the same forest, Schnabel et al. (2022)^24^ found strong decreases in tree growth and increased physiological stress after consecutive droughts, and there is ample evidence for negative effects of droughts on tree growth in other temperate floodplain forests, inducing increased stress and higher tree mortality^23,92,93^. Thus, it is likely that the compound event of ash dieback and climate extremes might cause the negative growth reactions observed on dry sites in our study.

With good water supply, however, increased light availability and warmer temperatures can be used to increase the rate of photosynthesis, as the transpiration losses can be quickly compensated for, which might explain the positive growth responses to ash dieback we observed on moist sites. Multiple studies found evidence for positive growth responses of residual trees to canopy disturbance^26,38,47,97^, and access to groundwater was found to buffer negative effects of summer drought on floodplain forest tree growth, at least in oak trees^25^. Furthermore, water from the groundwater is available for fine roots through capillary rise when the distance to groundwater is low^98^, and unpublished data from our forest ecosystem suggest that soil moisture is strongly correlated with distance to groundwater level (Vieweg et al. unpublished data), making distance to groundwater a good predictor for soil moisture. However, positive growth reactions on moist sites were generally quite weak suggesting that not enough time has passed yet to physiologically adapt to the new environmental conditions^35,39^. Alternatively, it is possible that trees may not be capable of utilizing improved resource availability because they are suffering from drought legacy effects of the 2018 and 2019 consecutive drought years^24,99^. It is important to note that our results represent the effects of a compound event of a pathogen invasion and climate extremes opening up the canopy via ash mortality, and the observed differences between moist and dry sites may reflect shifting climatic conditions. The effects we see on dry sites can actually be an indicator of reorganization processes under progressing climate change and canopy breakdown. It further has to be noted that, during our study period, the ash dieback was just at its beginnings and accelerated tremendously after 2020 ^67^, likely leading to even more pronounced effects over a longer observation period. Our result that growth responses depend on hydrological conditions is in line with our hypothesis but contrasts previous findings of a dendrochronological study in the Leipzig floodplain forest, which found only minor effects of hydrology on tree growth during the 2018–2019 consecutive drought^24^. This discrepancy may be due to differences in study methods, such as our more fine-scaled hydrological data^59^ and larger sample size.

### Growth responses in the lower and upper canopy

We hypothesized that the lower canopy would show stronger positive growth responses to ash dieback, given the greater increase in light availability, while the upper canopy would show weaker responses due to less light competition. Our results reveal seemingly different growth patterns for the lower and upper canopy. In the lower canopy, species-specific growth responses varied with both ash dieback intensity and hydrological conditions. However, contrary to our third hypothesis, we did not observe particularly pronounced positive growth reactions, but rather strong differences between moist and dry sites, especially for *A. campestre*, *A. pseudoplatanus*, and *C. betulus*. In the upper canopy, the effects of ash dieback and hydrological conditions were less pronounced, with no species-specific reactions. This could be due to the larger rooting depth of overstory trees, which are better able to buffer hydrological variations. Increasing canopy disturbance reduces the canopy’s ability to buffer against climate extremes^50,100–102^, exposing lower-canopy trees to stronger microclimatic fluctuations and more direct coupling with the atmosphere. This can result in reduced growth rates due to higher radiation, air temperature, and vapor pressure deficit (VPD)^103–105^. Larger trees in the upper canopy may also be less capable of responding to the increased resource availability in dependence on hydrology due to physiological constraints associated with age, as they are already longer adapted to their site conditions than smaller trees^35,43^.

The negative growth responses for lower canopy trees on dry sites are consistent with other studies showing that drought and competition can increase mortality, especially in understory trees^106^. Mortality is usually preceded by growth declines indicative of inciting stress that can span several decades^107–109^. Smaller trees may furthermore have limited access to deeper water layers due to their less developed root systems^110^ and may thus be more prone to drought-induced stress. This may explain the pronounced negative growth reactions in species like *C. betulus* and *T. cordata* on dry sites, which suffer disproportionally from the novel dry conditions as compared to other species, above all *Acer*. Our results suggest that if soil conditions continue to dry up with proceeding climate change, growth decline might ultimately lead to increased mortality for some species but not for others in the lower canopy and thus to a change in forest composition, structure and functioning.

Previous studies showed that smaller overstory trees show a relatively greater growth response to improved light access than larger trees, especially when they grow near a canopy gap^38,40,41^ (but see^44,111^). We did not find a particularly pronounced positive growth reaction to increased light availability in the lower canopy. It has to be noted, though, that as we could not integrate canopy layer as an interaction effect in our model due to an unbalanced representation of several species in both tree layers, we cannot directly test for different growth responses to ash dieback between the lower and upper canopy. What became evident, though, was that growth responses were in general higher in the lower as compared to the upper canopy, which is in line with previous findings showing that growth rates decline with increasing tree age and size^112^, but see^113^.

### Species-specific growth responses in the light of shade and drought tolerance

We expected growth responses to ash dieback and hydrological conditions to differ between species. Consistent with this hypothesis, we found species-specific growth responses to ash dieback and hydrological conditions in the lower canopy but not in the upper canopy where species in general showed differential growth responses but not in relation to ash dieback and distance to groundwater. Species-specific responses to varying environmental conditions can be explained by differences in shade or drought-tolerance and specific water demands. Although, according to Ellenberg’s ecological indicator values^114^, our examined tree species show similar levels of shade and drought tolerance, being intermediate for all species except for *Q. robur* which stands out due to its low shade tolerance and high drought tolerance^115^, there is some variation, with *A. campestre* and *T. cordata* being slightly more light demanding (L5) than the remaining species (L4) (see Table S13 Supplementary Material). *A. campestre* and *T. cordata*, the less shade-tolerant species, profited the least from canopy disturbance on moist sites in the lower canopy. *T. cordata* significantly differed from all other species in its reaction to ash dieback on moist sites, being the only species showing pronounced negative growth reactions when water availability was sufficient. Our results are in line with previous findings showing that more shade-tolerant species respond more positively to increased light availability due to canopy gaps than less shade-tolerant species, especially smaller individuals in the lower canopy^38^ (but see^47^). In general, shade-tolerant species are more likely to respond to small gap openings as their physiological and morphological plasticity enables them to quickly adjust to increased light availability^36^, which might explain the more positive growth responses of more shade-tolerant species on moist sites in our study. However, it has to be acknowledged that the pattern we found on moist sites is subtle and that the distinct negative reactions of *T. cordata* could also be attributed to high density competition by *Acer* species, especially *A. pseudoplatanus*. Furthermore, Ellenberg’s classification may not fully capture the variation in growth responses related to shade and drought tolerances as it does not reflect physiological or competitive dynamics. More quantitative functional traits, such as leaf area ratio^116^ or xylem potential (P50^54^), may help to better understand the underlying mechanisms of the observed pattern and could serve as the focus of future analyses.

On dry sites, *C. betulus* showed the most pronounced negative growth response to ash dieback intensity, which differed significantly from growth responses of all other species except *T. cordata*. This finding is somewhat surprising, as *C. betulus* is thought to have a moderate to high level of drought tolerance in the adult stage^115^ (see Table S13 Supplementary Material) and high drought resistance in the juvenile stage^117^ that is not below that of the other species. The negative reactions might most likely be the result of indirect competitive interactions between the species, especially with *A. pseudoplatanus*, as are the pronounced negative responses of *T. cordata* on both moist and dry sites. Demographic data from our two inventories show that *A. pseudoplatanus* is highly abundant, both in the regeneration layer and the lower canopy, that its abundance increased over the study period and that it shows very high recruitment^21,118,119^ (Figs. 1a and 2b, Fig. S7 Supplementary Material). In the regeneration layer, *Acer* species and above all *A. pseudoplatanus* showed the strongest positive growth responses to increased canopy openness compared to other species^120^. Thus, although we cannot confirm this based on the results of our model, it is well conceivable that *A. pseudoplatanus* might outcompete other species in the lower canopy because of fast recruitment and high abundance. The high growth increments of *A. pseudoplatanus* observed in advanced regeneration (in the lower DBH classes from 5 to 40 cm, see Fig. S6 Supplementary Material) indicate a shift in forest development towards this competitive species that could result in progressing competitive takeover, especially if hydrological conditions worsen in the light of climate change, favoring the flood-intolerant *A. pseudoplatanus*^121^ even more. Besides, we observed a general competition effect on growth (Fig. S12, Supplementary Material). Due to model complexity, we were unable to include the four-way interaction between species, ash dieback, hydrology, and plot density. Future studies could explore these interaction effects in more detail. Furthermore, intense competition among smaller trees in the lower canopy may not be fully captured by basal area alone. Incorporating the number of individuals in future analyses could provide a more comprehensive understanding of competition effects.

*Ulmus* seemed to be unaffected by ash dieback in the lower canopy, both under moist and dry hydrological conditions, which is likely due to its vegetative reproduction mode that makes them less sensitive to changing and adverse environmental conditions^122^. Elm trees are basically non-existent anymore in the upper canopy due to the Dutch elm disease which increasingly spread in the 1960s, but are still abundant in the regeneration layer and lower canopy^21,118^ (see Fig. 1). Although most *Ulmus* species will most likely not play a role in the upper canopy anymore as trees die when reaching the adult phase due to the fungal disease, our findings suggest that *Ulmus* is likely to persist in the lower canopy even under adverse novel environmental circumstances.

It would have been interesting to see how *Q. robur* as the most light-demanding species (L7) in our sample reacts to increased light availability in the lower canopy. However, *Q. robur* is hardly present in the lower canopy and almost absent in the regeneration layer^21,118,123,124^ (see Fig. 1c&d), as it is no longer able to successfully regenerate under natural circumstances, mostly due to the limited availability of light on the forest floor which is exacerbated by the dominating spread of shade­casting *Acer* species^33,55,73^ (see Fig. S7 Supplementary Material). We could therefore not reliably model its growth responses to increased light availability in the lower canopy. In the upper canopy, *Q. robur* did not seem to perform any worse or better to increased light availability compared to the other species, although growth rates seemed to be generally lower in *Q. robur* than in other species (see Fig. 2; Table 2). It is important to note that the current high proportion of oak in the upper canopy is partly a legacy of historical management practices, such as coppicing-with-standards^21^. However, due to its inability to regenerate successfully and the ongoing mortality of older trees, *Q. robur* is at risk of gradually disappearing from the hardwood floodplain forest ecosystem over time. From an ecological and conservation perspective, *Q. robur* is of utmost importance as it supports a very high and specific biodiversity for a wide range of species groups^125–128^ and is particularly vital for many threatened species^129,130^. Thus, its preservation is necessary for the ecosystem’s conservation status and requires targeted silvicultural interventions. Small clear-cuts – similar to the femel cuttings currently in practice – could create favorable conditions for light-demanding oaks and promote their continued presence in the ecosystem^73,131^.

### Implications for conservation

Floodplain forests worldwide show an unfavourable to bad conservation status^33,132^, threatening their role as biodiversity hotspots^13,15^ and the numerous ecosystem functions they provide^16,17,133,134^, and are thus highly protected on a global and local scale^13,55^. Even small changes in the water dynamics can lead to a drastically altered species composition and diversity^13^. Our results emphasize the role of groundwater levels in influencing tree growth responses to canopy disturbances caused by ash dieback. High groundwater levels appear to mitigate negative effects of canopy disturbances on residual tree growth, highlighting the importance of revitalizing hydrodynamics and raising groundwater levels in floodplain forest ecosystems in the face of extreme drought events. According to our findings, *Acer* species —particularly *A. pseudoplatanus* and *A. platanoides*— are likely to dominate future floodplain forests, as they exhibit high growth rates that decline less under dry and light conditions compared to species like *C. betulus* or *T. cordata*. Our results also suggest that if there will only be a moderate degree of rewetting without a substantial revitalization of the floodplain, this may inadvertently favor *A. pseudoplatanus* instead of displacing it. The duration of rewetting must be sufficient to selectively increase mortality substantially for flood-intolerant^121^ *Acer* species, thus removing them from the system and release other species from density competition by *Acer*.

Thus, if revitalization measures are not being implemented soon enough, the dominance of *Acer* species is likely promoted. With the loss of *Ulmus* and now *F. excelsior* and the continuous decline of *Q. robur* within the past decades, the ecosystem might lose its three characteristic main tree species and with them countless threatened species that depend on them. As a consequence, the conservation status of hardwood floodplain forests as Natura 2000 site is threatened. As *Acer* species are likely to reach lower heights than *F. excelsior*, an *Acer* dominated ecosystem could result in reduced biomass, smaller ecosystem volume, and less carbon sequestration. The continued darkening of the forest floor by *Acer* would potentially furthermore alter the summer aspect of the ground vegetation. If revitalization measures would then be implemented over time, the flood-intolerant *Acer* may not be able to adapt to the restored hydrodynamics, which could, in the worst-case scenario, lead to a transient collapse of the overstorey.

Alternatively, if revitalization measures, such as raising riverbeds, reactivating and integrating antiquated watercourses, dismantling or perforating dikes^34^, will be implemented soon enough and restore natural flooding dynamics and elevated groundwater levels, together with nature conservation-compliant promotion of oak regeneration, the hardwood forest community might have a chance to maintain a state that at least resembles the characteristic composition. Although we found that the intensity of ash dieback does not seem to depend on hydrological conditions (see Fig. S9c Supplementary Material) and thus revitalization might not stop or ameliorate the disease, resistant genotypes or epigenetic responses might keep ash trees in the system in the medium term^135–137^. Our results indicate that *Acer* might curtail its dominance together with an increased mortality due to the sooty bark disease (*Cryptostroma corticale*)^21,73^. A reestablished natural flooding regime might furthermore suppress *Acer* regeneration. Areas of increased mortality and thus increased light availability could be used to actively promote the light-demanding *Q. robur* through planting and removal of *Acer* species, as it was shown that the vitality of *Q. robur* saplings is highest under these conditions (Lenk et al. unpublished data).

## Conclusion and outlook

Our study looked at growth as one component of forest reorganization processes and focused on early responses after the canopy has just begun to open up due to ash dieback. Monitoring these initial effects provides valuable insights into species’ growth before competitive interactions dominate. However, it is important to note that reorganization not only depends on growth, but also on other demographic rates such as mortality and recruitment that we did not consider here. In order to fully understand the mechanisms of forest reorganization processes, it is of utmost importance to continue the observations and to include all demographic rates. Currently, efforts are made to model these dynamics in more detail using field-parameterized demographic growth models^138^. Furthermore, we observed responses to canopy release even over a relatively short time interval. These effects may become more pronounced over time, and species not yet affected could potentially be impacted in the long term.

Our study’s findings for moist and dry site conditions could reflect “past/normal” and “future/novel” hydrological states. The negative effects on dry sites might even underestimate the impact of climate change and increasing droughts, as trees on moist sites may suffer even more from sudden hydrological changes. Thus, our results most likely represent a rather conservative estimation. If abiotic conditions change substantially in the future, previously absent or underrepresented species, such as the European beech, may establish in the system, altering forest composition and functioning. Findings from this study and related research could help predict forest responses to changing climate conditions.

## Electronic supplementary material

Below is the link to the electronic supplementary material.

Supplementary Material 1

## Data Availability

Tree inventory data are available at https://doi.pangaea.de/10.1594/. Ash dieback inventory data are currently under review at Pangaea and will be available at https://doi.pangaea.de/10.1594/PANGAEA.977358.  R-scripts and functions are available upon reasonable request. Please contact Stefanie Henkel at shenkel@uni-leipzig.de.
