# Supplementary material for: Ash dieback and hydrology affect tree growth patterns under climate change in European floodplain forests
Source: Sci Rep. 2025 Mar 24;15:10117. doi: 10.1038/s41598-025-92079-5 (PMC11933702; doi:10.1038/s41598-025-92079-5)
Supplement: Supplementary file 1 — Supplementary Material 1 [file 41598_2025_92079_MOESM1_ESM.pdf]

Supplementary Material

Ash dieback and hydrology affect tree growth patterns under climate change in European floodplain forests

Stefanie Henkel*, Ronny Richter, Karl Andraczek, Roger Mundry, Madeleine Dontschev, Rolf A. Engelmann, Timo Hartmann, Christian Hecht, Hans Dieter Kasperidus, Georg Rieland, Mathias Scholz, Carolin Seele-Dilbat, Michael Vieweg, Christian Wirth

*Corresponding author: shenkel@uni-leipzig.de

Table S1 Number of individuals per species in the first and second inventory**.**

**Species**	**First inventory**	**Second inventory**	
Acer campestre L.	432	527	↑
Acer platanoides L.	470	561	↑
Acer pseudoplatanus L.	1.305	1.358	↑
Aesculus hippocastanum L.	23	23	-
Alnus glutinosa (L.) GAERTN.	28	28	-
Betula pendula ROTH	7	6	↓
Carpinus betulus L.	599	623	↑
Corylus avellana L.	111	131	↑
Cornus sanguinea L.	6	16	↑
Crataegus spec.	70	75	↑
Euonymus europaeus L.	0	1	↑
Fagus sylvatica L.	26	27	↑
Fraxinus excelsior L.	1.037	838	↓
Fraxinus pennsylvanica MARSHALL	11	7	↓
Larix decidua MILL.	1	1	-
Malus sylvestris MILL.	8	11	↑
Populus spec.	24	22	↓
Prunus avium MILL.	1	2	↑
Prunus padus L.	15	32	↑
Quercus robur L.	438	381	↓
Quercus rubra L.	18	17	↓
Rhamnus cathartica L.	0	1	↑

Robinia pseudoacacia L.	6	6	-
Sambucus nigra L.	253	343	↑
Tilia cordata MILL.	749	943	↑
Tilia platyphyllus SCOP.	44	94	↑
Ulmus spec.	1.457	1.536	↑
**Total**	**7.139**	**7.610**	

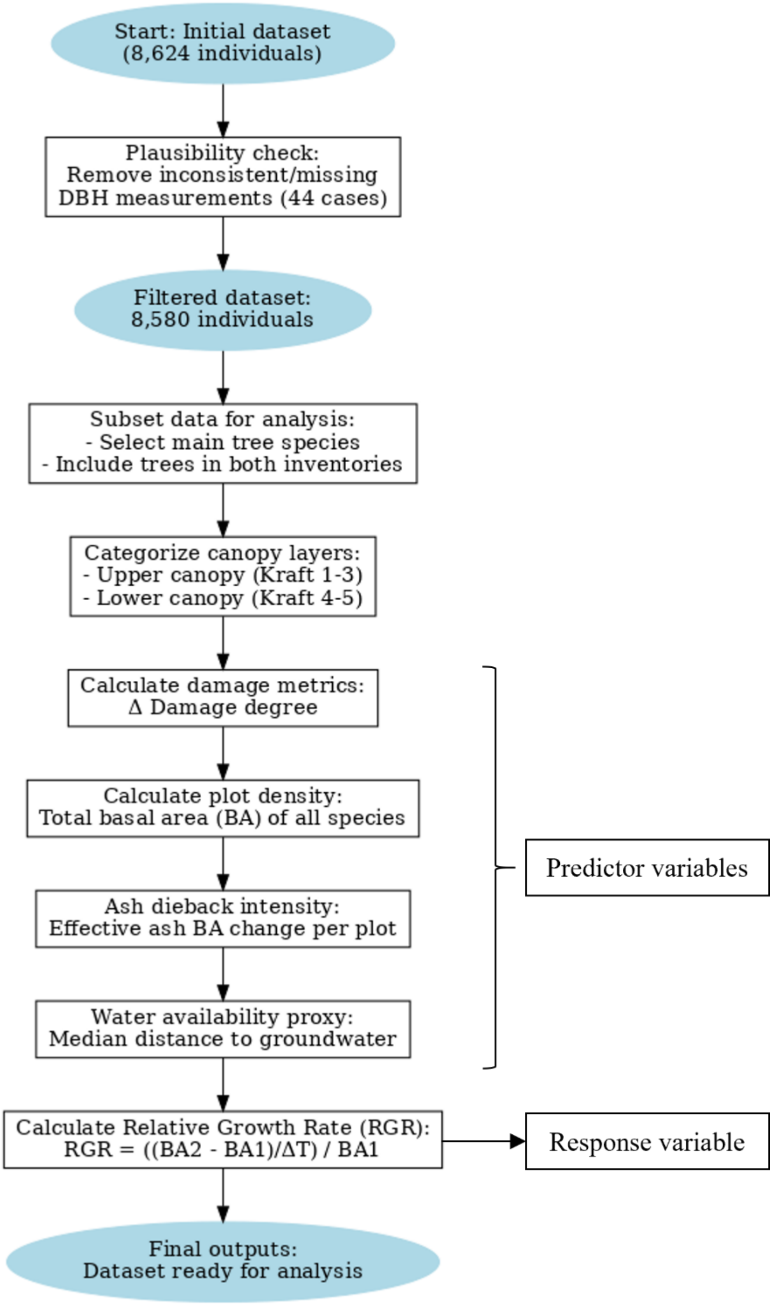

Fig. S1 Flowchart summarizing the data preparation workflow.

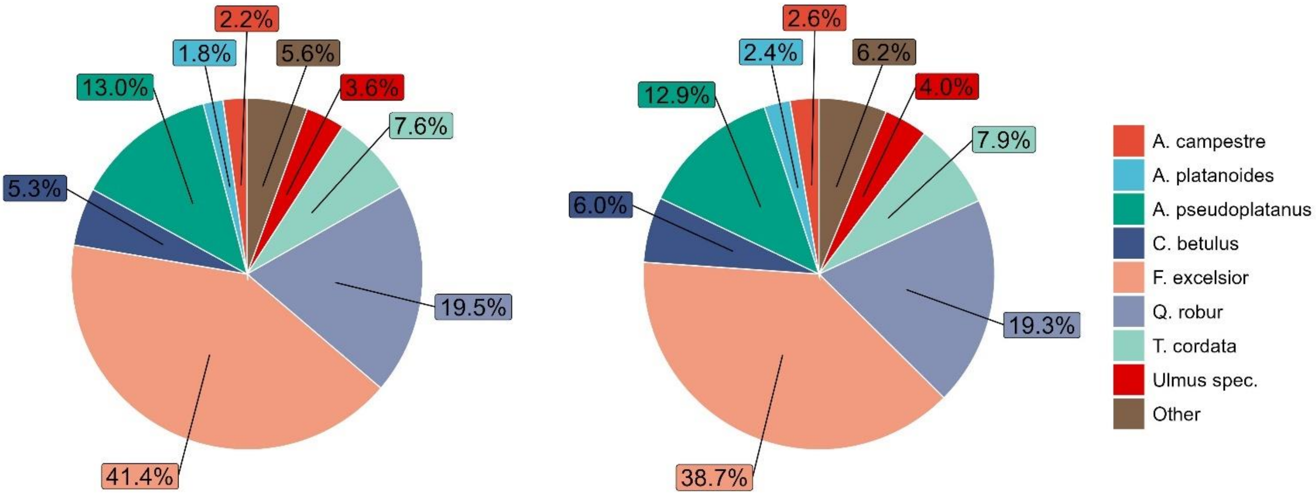

Fig S2. Species composition (relative dominance) in the tree layer (DBH ≥ 5 cm) on the 60 study plots in the Leipzig floodplain forest in the first (left) and second inventory (right). Relative dominance is related to basal area. First inventory: N = 7139, second inventory: N = 7610.

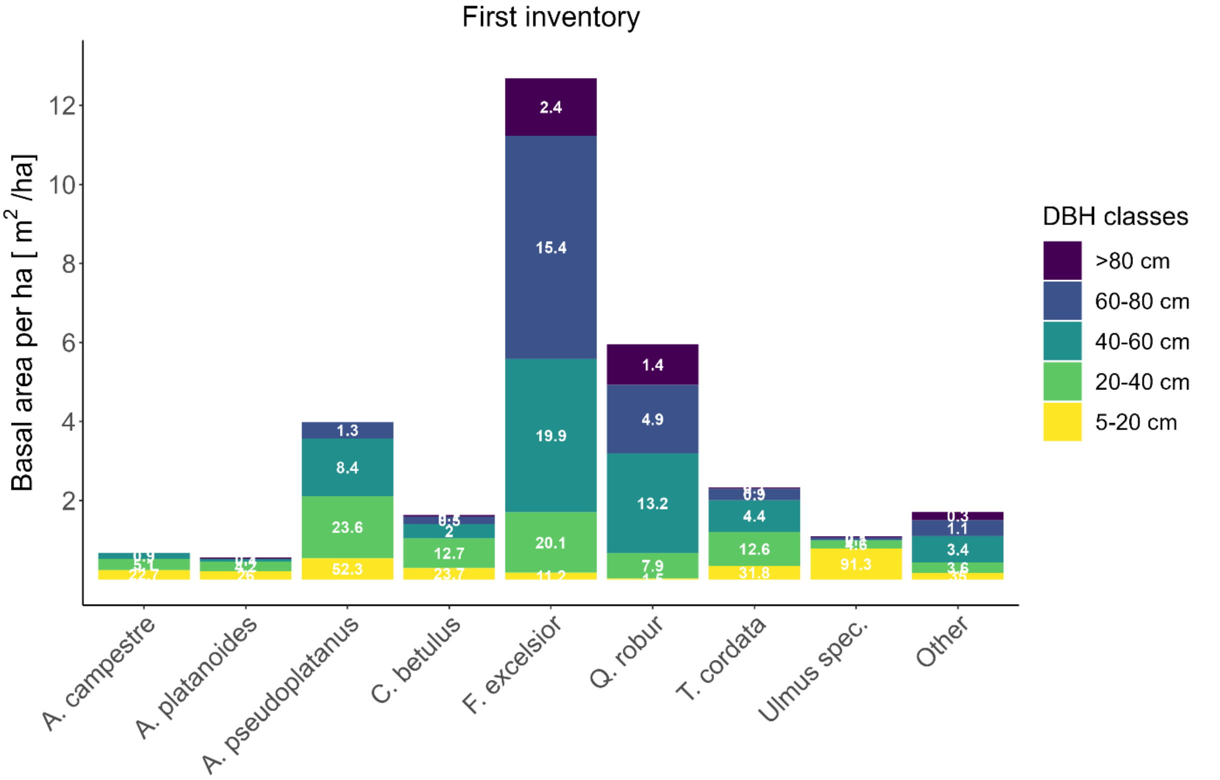

Fig S3. Basal area per tree species and diameter classes on the 60 study plots in the Leipzig floodplain forest in the first inventory. The numbers within the bars indicate the number of individuals per hectare.

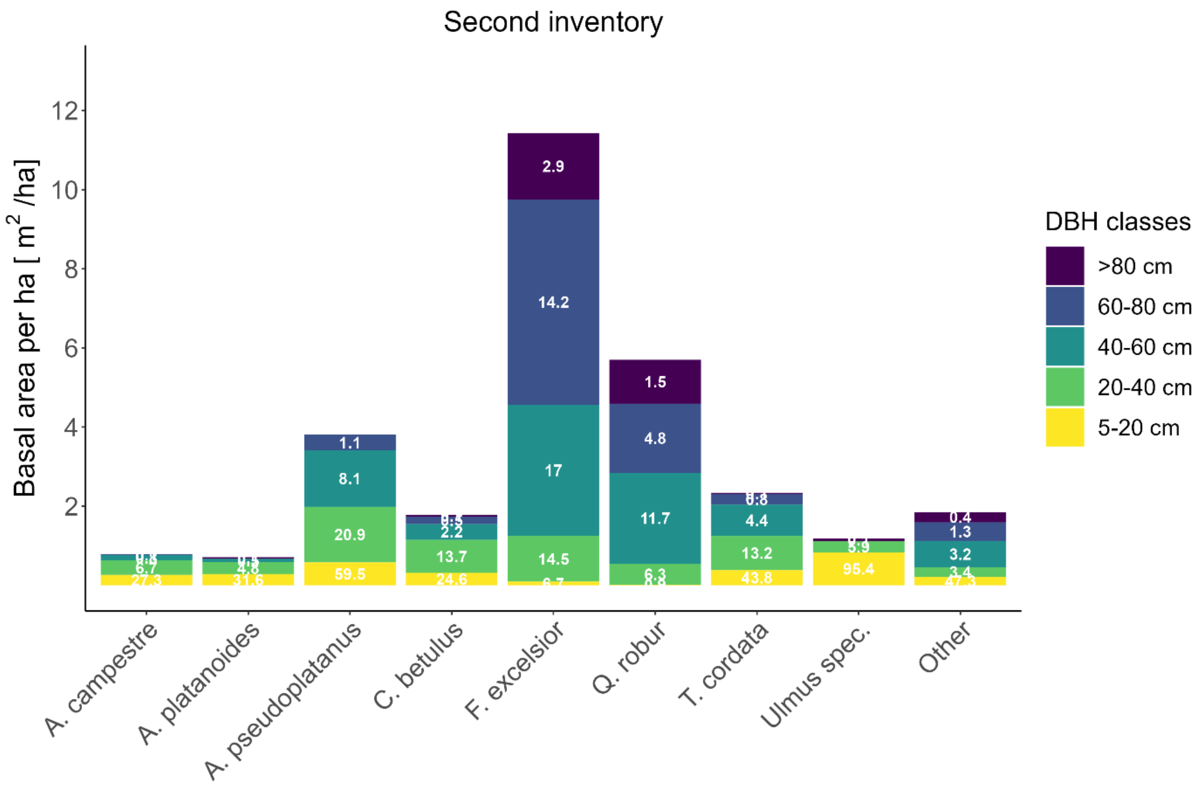

Fig S4. Basal area per tree species and diameter classes on the 60 study plots in the Leipzig floodplain forest in the second inventory. The numbers within the bars indicate the number of individuals per hectare.

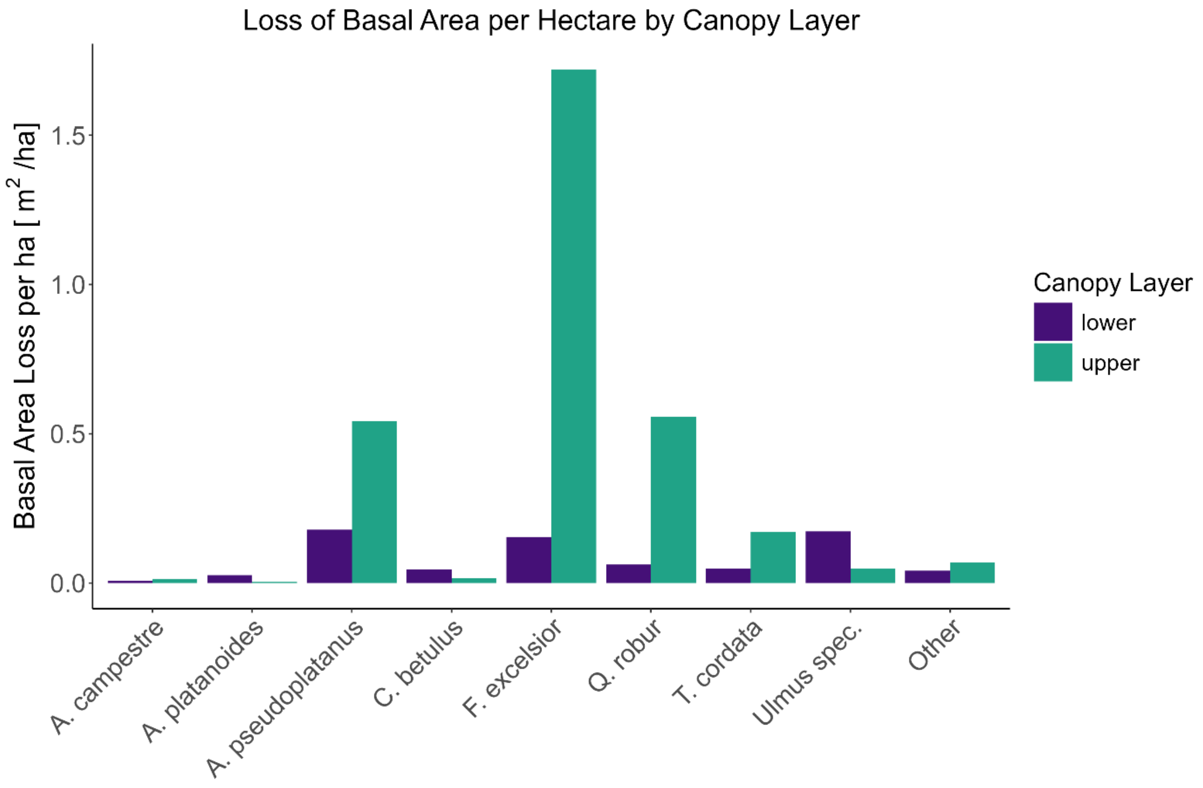

Fig. S5. Losses in basal area per tree species and canopy layer on the 60 study plots in the Leipzig floodplain forest.

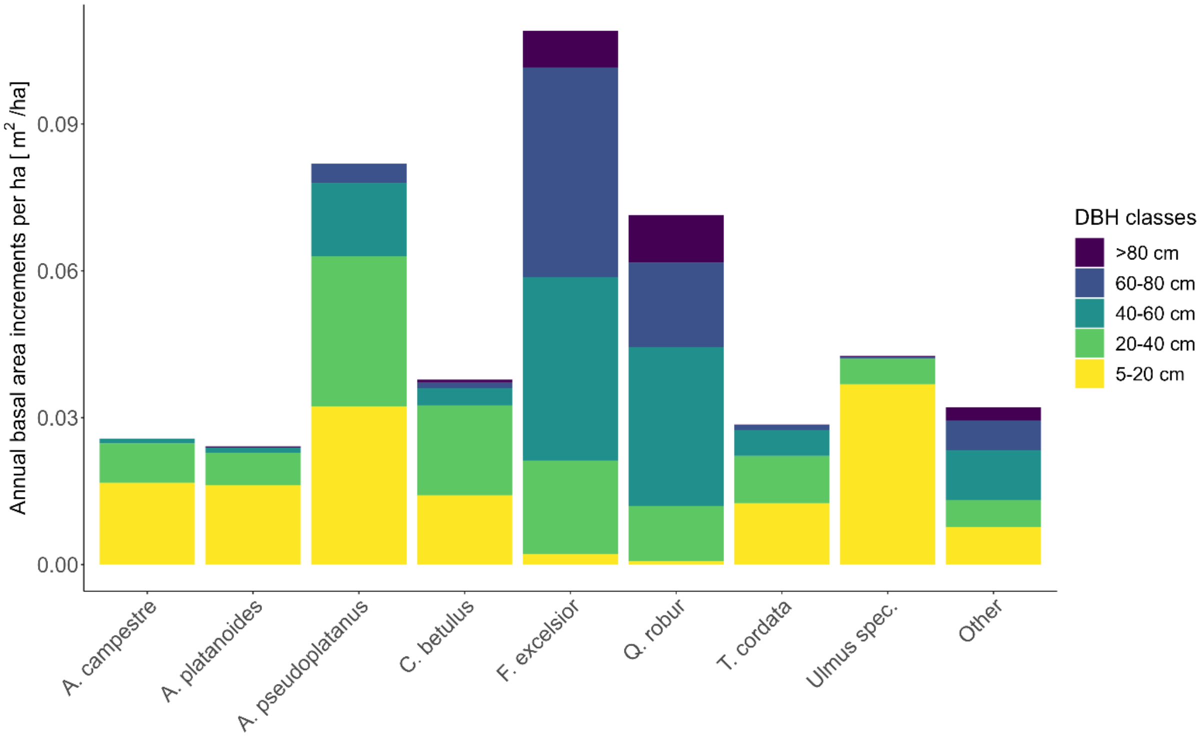

Fig. S6. Distribution of basal area increments across tree species and diameter classes on the 60 study plots in the Leipzig floodplain forest.

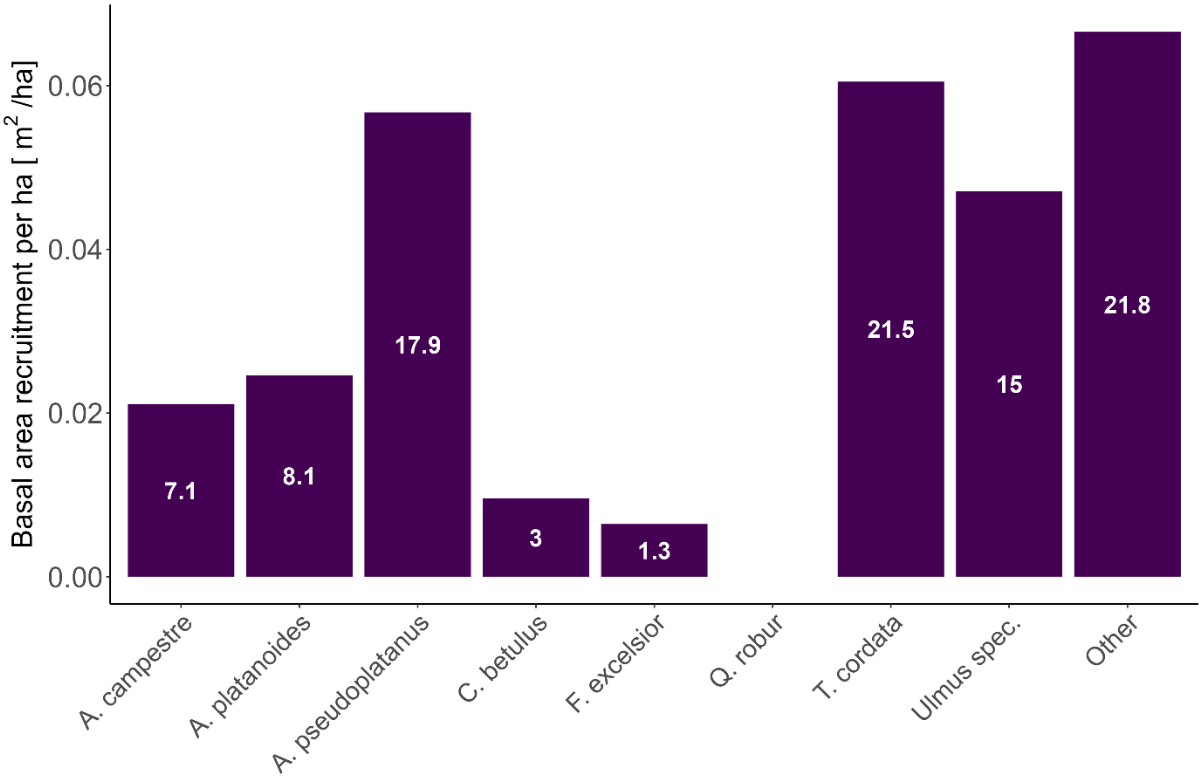

Fig. S7. Basal area of recruitment per tree species on the 60 study plots in the Leipzig floodplain forest. Recruitment refers to the total basal area of those individuals that reached the threshold of 5cm diameter at breast height (DBH) from the first to the second inventory. The numbers within the bars indicate the number of individuals per hectare.

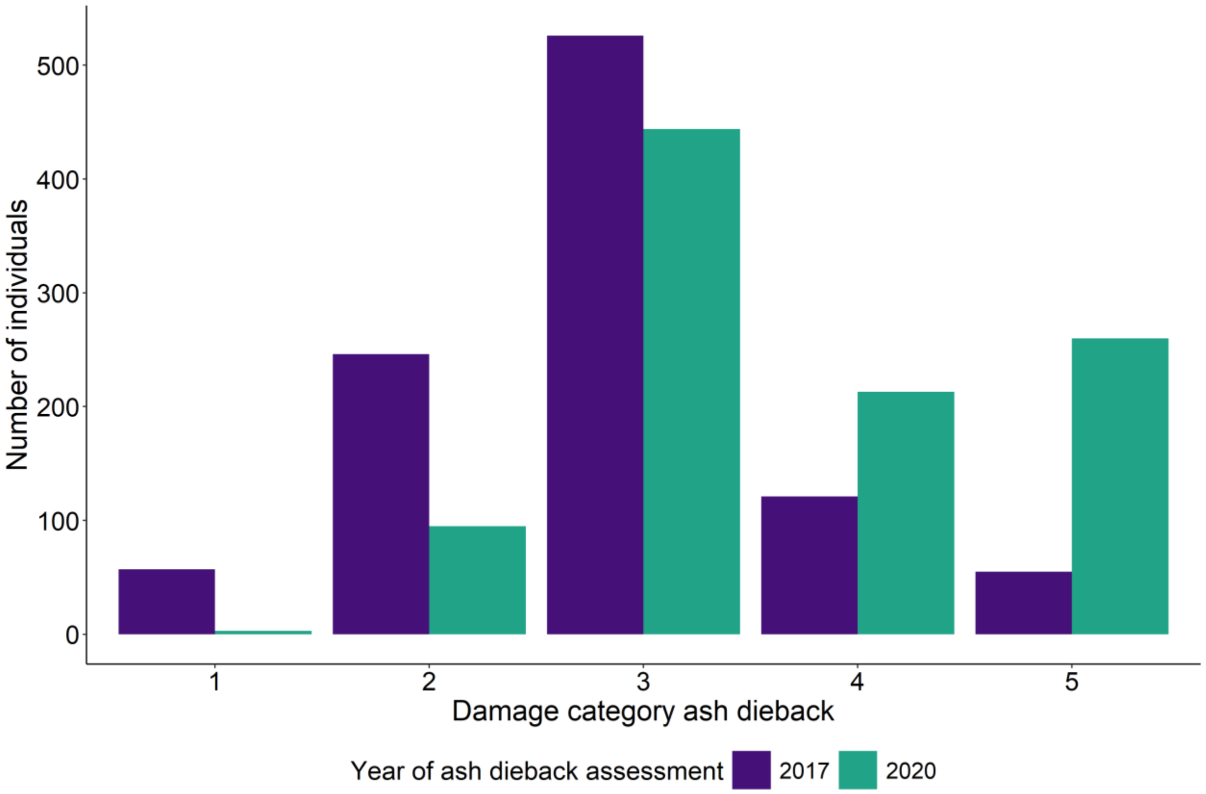

Fig. S8. Extent of ash dieback in the Leipzig floodplain forest according to the ash dieback inventory for the years 2017 and 2020. The figure shows the number of all ashes (n = 1001 for both years) per ash dieback damage category according to Lenz et al. (2012). Subdominant individuals were not included. Note that there are no vital ashes (category = 0) left.

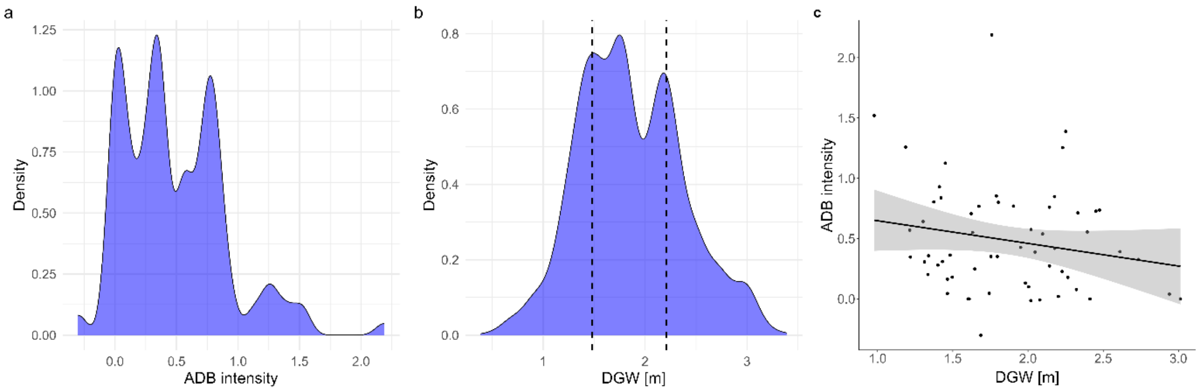

Fig. S9 a. Distribution of ash dieback (ADB) intensity. **b.** Distribution of distance to ground water (DGW). Dashed vertical lines indicate the 25% and 75% quantiles. **c.** Correlation between ADB and DGW (Pearson's correlation: r= -0.192, t= -1.476, df=57, p= 0.146).

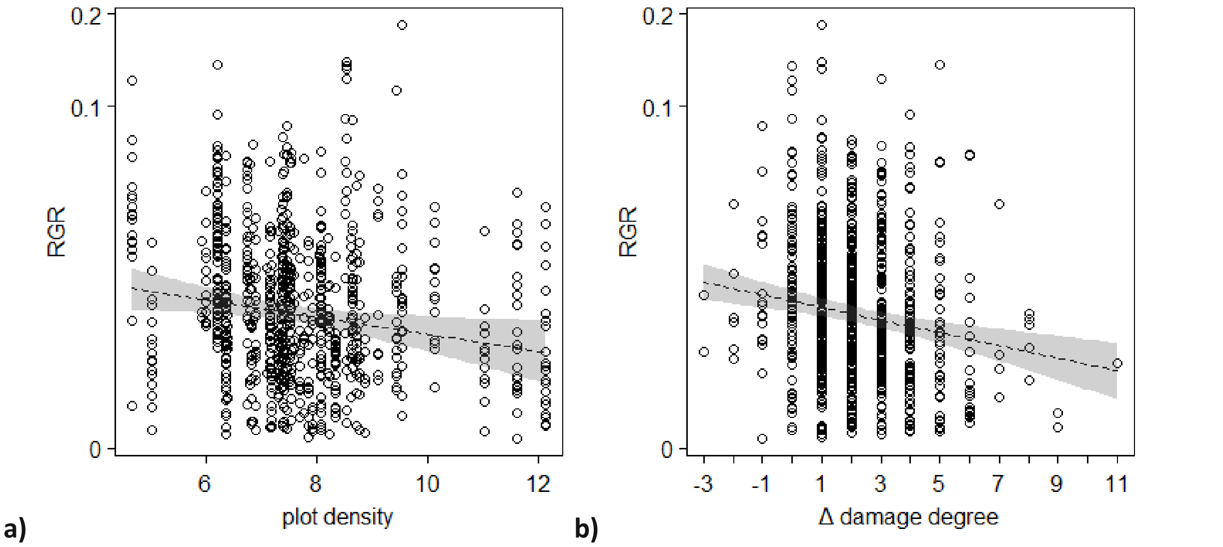

Figure S10 Effect of a) plot density and b) Δdamage degree on relative growth rates in the upper canopy. Dashed lines and grey polygons depict the fitted model and its 95 % confidence limits.

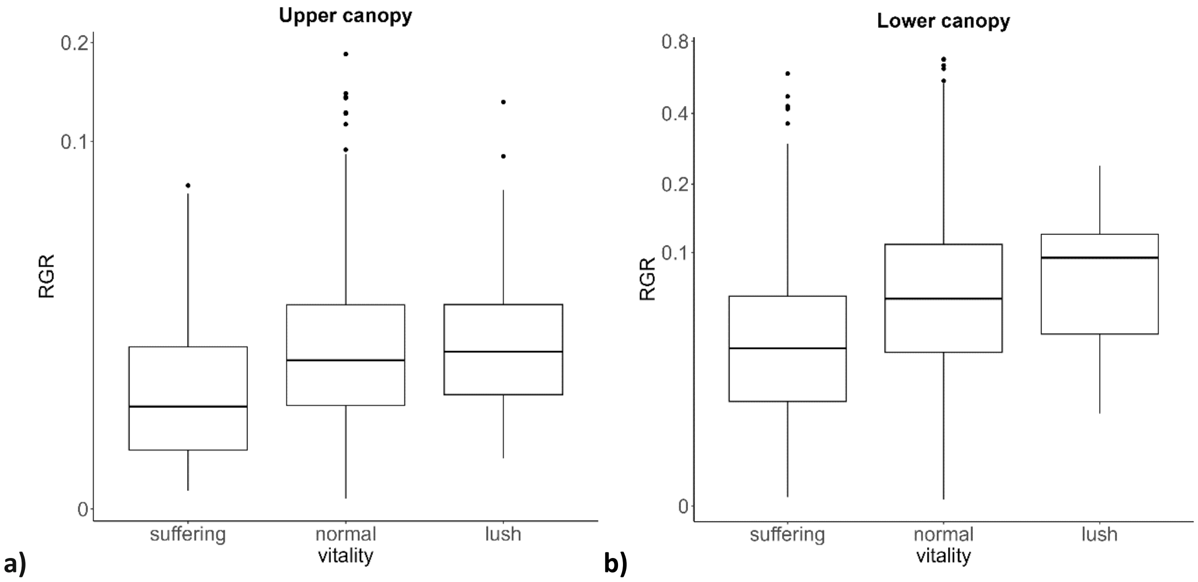

Figure S11 Effect of tree vitality on relative growth rates (RGR) in a) the upper canopy and b) the lower canopy.

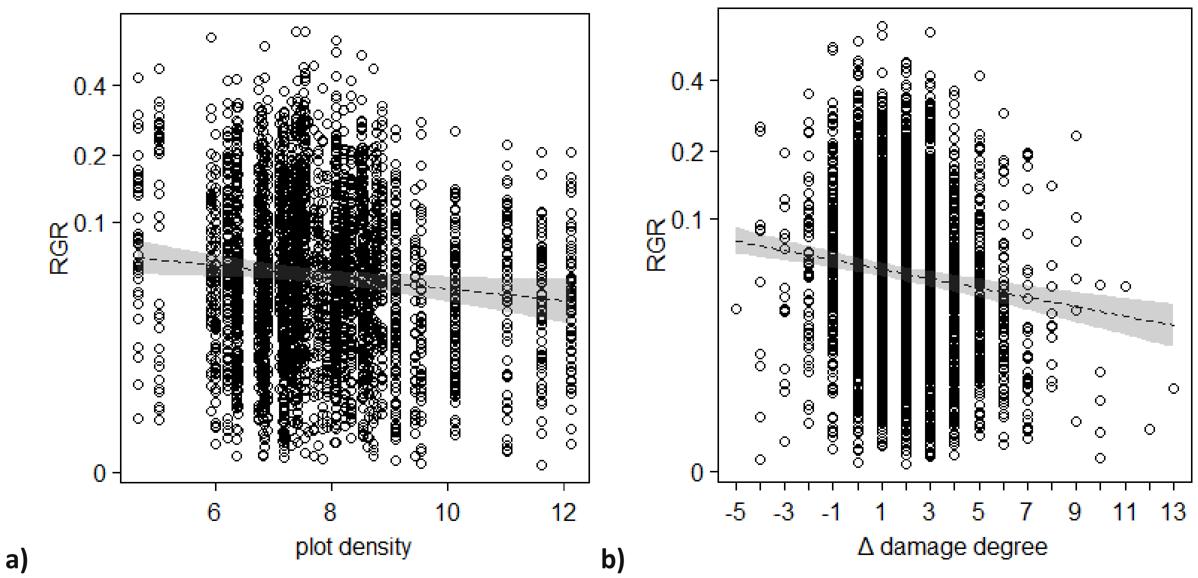

Figure S12 Effect of a) plot density and b) Δdamage degree on relative growth rates in the lower canopy. Dashed lines and grey polygons depict the fitted model and its 95 % confidence limits.

Table S2 Results of the linear mixed model (LMM) testing for species-specific differences between the lower and upper canopy. Ace_cam= *Acer campestre*, Ace_pla= *Acer platanoides*, Ace_pse= *Acer pseudoplatanus*, Car_bet= *Carpinus betulus*, Que_rob= *Quercus robur*, Til_cor= *Tilia cordata,* Ulm_spe= *Ulmus spec.*; spec= species.

Term	Estimate	SE
Intercept	9.10E-02	4.47E-03
canopy_layer.upper	-5.65E-02	1.36E-02
spec.Ace_pla	1.65E-02	4.98E-03
spec.Ace_pse	-1.54E-02	4.24E-03
spec.Car_bet	-3.24E-02	4.56E-03
spec.Que_rob	-7.91E-02	1.17E-02
spec.Til_cor	-3.81E-02	4.54E-03
spec.Ulm_spe	-2.00E-02	3.96E-03
canopy_layer.upper:spec.Ace_pla	-1.15E-02	1.69E-02
canopy_layer.upper:spec.Ace_pse	2.95E-03	1.43E-02
canopy_layer.upper:spec.Car_bet	2.25E-02	1.57E-02
canopy_layer.upper:spec.Que_rob	5.87E-02	1.80E-02
canopy_layer.upper:spec.Til_cor	1.87E-02	1.48E-02
canopy_layer.upper:spec.Ulm_spe	4.18E-02	1.84E-02

Table S3 Results of the pairwise post-hoc comparison between lower and upper canopy per species. Indicated are estimates, standard errors (SE), degrees of freedom, the t-ration and p-values. Ace_cam= *Acer campestre*, Ace_pla= *Acer platanoides*, Ace_pse= *Acer pseudoplatanus*, Car_bet= *Carpinus betulus*, Que_rob= *Quercus robur*, Til_cor= *Tilia cordata,* Ulm_spe= *Ulmus spec*.; spec= species.

Contrast	Esti-mate	SE	df	t-ratio	p
lower vs. upper (Ace_cam)	0.057	0.014	4665	4.147	<0.0001
lower vs. upper (Ace_pla)	0.068	0.010	4671	6.793	<0.0001
lower vs. upper (Ace_pse)	0.054	0.004	4708	12.067	<0.0001
lower vs. upper (Car_bet)	0.034	0.008	4691	4.443	<0.0001
lower vs. upper (Que_rob)	-0.002	0.012	4674	-0.182	0.856
lower vs. upper (Til_cor)	0.038	0.006	4689	6.751	<0.0001
lower vs. upper (Ulm_spe)	0.015	0.012	4676	1.190	0.234

Table S4 Results of the full linear mixed model (LMM) for the upper canopy. Indicated are estimates, standard errors (SE), numerator and denominator degrees of freedom (Num df and Den df), significance tests, confidence limits, and the range of estimates obtained when dropping levels of grouping factors one at a time. Ace_pse= *Acer pseudoplatanus*, Car_bet= *Carpinus betulus*, Que_rob= *Quercus robur*, Til_cor= *Tilia cordata*; spec= species; ADB= ash dieback, as effective basal area change of ash trees; DGW = distance to ground water; density= plot density; Δdamage= delta damage degree; ΔT= time interval between first and second inventory.

Term	Estimate	SE	F	Num df	Den df	p	lower CI	upper CI	min	max
Intercept	-3.468	0.104	(1)	(1)	(1)	(1)	-3.664	-3.268	-3.600	-3.424
spec.Ace_pse(2)	-0.206	0.106	(1)	(1)	(1)	(1)	-0.403	0.008	-0.244	-0.054
spec.Car_bet(2)	-0.412	0.140	(1)	(1)	(1)	(1)	-0.606	-0.057	-0.514	-0.217
spec.Que_rob(2)	-0.456	0.105	(1)	(1)	(1)	(1)	-0.661	-0.283	-0.514	-0.326
spec.Til_cor(2)	-0.518	0.107	(1)	(1)	(1)	(1)	-0.709	-0.285	-0.550	-0.375
ADB(3)	-0.069	0.133	(1)	(1)	(1)	(1)	-0.213	0.305	-0.063	0.129
DGW(3)	-0.119	0.105	(1)	(1)	(1)	(1)	-0.386	0.063	-0.303	-0.068
density(3)	-0.076	0.033	5.452	1	47.230	**0.024**	-0.159	-0.019	-0.106	-0.062

Δdamage(3)	-0.063	0.017	13.867	1	777.590	**<0.001**	-0.112	-0.039	-0.083	-0.068
vitality.normal(4)	0.189	0.060	4.932	2	46.320	**0.011**	0.054	0.288	0.136	0.188
vitality.vital(4)	0.268	0.133	(1)	(1)	(1)	(1)	-0.022	0.522	0.158	0.279
ΔT(3)	0.014	0.032	0.201	1	52.540	0.655	-0.064	0.055	-0.025	0.015
spec.Ace_pse:ADB	0.019	0.141	(1)	(1)	(1)	(1)	-0.377	0.160	-0.194	-0.004
spec.Car_bet:ADB	0.047	0.181	(1)	(1)	(1)	(1)	-0.398	0.307	-0.166	0.049
spec.Que_rob:ADB	0.045	0.137	(1)	(1)	(1)	(1)	-0.310	0.191	-0.151	0.048
spec.Til_cor:ADB	0.037	0.144	(1)	(1)	(1)	(1)	-0.383	0.189	-0.191	0.013
spec.Ace_pse:DGW	0.088	0.113	(1)	(1)	(1)	(1)	-0.085	0.383	0.038	0.269
spec.Car_bet:DGW	-0.013	0.148	(1)	(1)	(1)	(1)	-0.216	0.402	-0.095	0.221
spec.Que_rob:DGW	0.167	0.113	(1)	(1)	(1)	(1)	-0.018	0.433	0.089	0.347
spec.Til_cor:DGW	0.086	0.116	(1)	(1)	(1)	(1)	-0.085	0.385	0.043	0.269
ADB:DGW	-0.135	0.149	(1)	(1)	(1)	(1)	-0.327	0.310	-0.123	0.082
spec.Ace_pse:ADB:DGW(5)	0.049	0.155	0.194	4	55.110	0.940	-0.356	0.264	-0.129	0.055
spec.Car_bet:ADB:DGW	0.041	0.213	(1)	(1)	(1)	(1)	-0.515	0.360	-0.187	0.049
spec.Que_rob:ADB:DGW	0.022	0.154	(1)	(1)	(1)	(1)	-0.399	0.219	-0.173	0.049
spec.Til_cor:ADB:DGW	0.074	0.162	(1)	(1)	(1)	(1)	-0.440	0.219	-0.185	0.026

(1): not shown because of being of very limited interpretability since term is involved in a higher order interaction
(2): species was dummy coded with Ace_pla being the reference level
(3): z-transformed to mean = 0 and sd = 1; mean and sd of the original variables are presented in table S5
(4): vitality was dummy coded with suffering being the reference level; the indicated significance test refers to the overall test of vitality
(5): the indicated significance test refers to the overall test of the 3-way interaction species:ADB:DGW

Table S5 Results of the reduced linear mixed model (LMM) for the upper canopy. Indicated are estimates, standard errors (SE), significance tests, confidence limits and the range of estimates obtained when dropping levels of grouping factors one at a time. Ace_pse= *Acer pseudoplatanus*, Car_bet= *Carpinus betulus*, Que_rob= *Quercus robur*, Til_cor= *Tilia cordata*; spec= species; ADB= ash dieback, as effective basal area change of ash trees; DGW = distance to ground water; density= plot density; Δdamage= delta damage degree; ΔT= time interval between first and second inventory.

Term	Estimate	SE	F	Num df	Den df	p	lower CI	upper CI	min	max
Intercept	-3.476	0.102	(1)	(1)	(1)	(1)	-3.662	-3.267	-3.596	-3.420
spec.Ace_pse(2)	-0.188	0.108	(1)	(1)	(1)	(1)	-0.394	0.019	-0.241	-0.057
spec.Car_bet(2)	-0.330	0.139	(1)	(1)	(1)	(1)	-0.601	-0.067	-0.454	-0.221
spec.Que_rob(2)	-0.449	0.101	(1)	(1)	(1)	(1)	-0.671	-0.267	-0.515	-0.327
spec.Til_cor(2)	-0.479	0.106	(1)	(1)	(1)	(1)	-0.697	-0.295	-0.549	-0.378
ADB(3)	-0.009	0.108	(1)	(1)	(1)	(1)	-0.229	0.235	-0.056	0.062
DGW(3)	-0.168	0.112	(1)	(1)	(1)	(1)	-0.368	0.096	-0.293	-0.065
density(3)	-0.085	0.032	6.870	1	27.674	**0.014**	-0.153	-0.019	-0.101	-0.063
Δdamage(3)	-0.075	0.019	16.053	1	57.185	**<0.001**	-0.112	-0.037	-0.083	-0.067
vitality.normal(4)	0.165	0.059	4.030	2	31.160	**0.028**	0.048	0.275	0.137	0.188
vitality.vital(4)	0.242	0.131	(1)	(1)	(1)	(1)	-0.035	0.490	0.163	0.282
ΔT(3)	-0.004	0.030	0.020	1	32.163	0.889	-0.067	0.057	-0.021	0.016
spec.Ace_pse:ADB(5)	-0.070	0.117	0.397	4	40.775	0.810	-0.317	0.163	-0.140	-0.023
spec.Car_bet:ADB	-0.009	0.133	(1)	(1)	(1)	(1)	-0.285	0.260	-0.081	0.037
spec.Que_rob:ADB	-0.006	0.111	(1)	(1)	(1)	(1)	-0.237	0.220	-0.080	0.042
spec.Til_cor:ADB	-0.040	0.120	(1)	(1)	(1)	(1)	-0.297	0.207	-0.112	0.012
spec.Ace_pse:DGW(6)	0.137	0.115	1.217	4	38.397	0.320	-0.147	0.349	0.033	0.261
spec.Car_bet:DGW	0.072	0.149	(1)	(1)	(1)	(1)	-0.243	0.363	-0.050	0.216
spec.Que_rob:DGW	0.200	0.114	(1)	(1)	(1)	(1)	-0.072	0.401	0.097	0.338
spec.Til_cor:DGW	0.143	0.119	(1)	(1)	(1)	(1)	-0.142	0.363	0.037	0.254
ADB:DGW	-0.083	0.030	7.929	1	27.955	**0.009**	-0.147	-0.023	-0.100	-0.068

(1): not shown because of being of very limited interpretability since term is involved in a higher order interaction
(2): species was dummy coded with Ace_pla being the reference level
(3): z-transformed to mean = 0 and sd = 1; mean and sd of the original variables are presented in table S5
(4): vitality was dummy coded with suffering being the reference level
(5): indicated significance test refers to the overall test of the interaction species:ADB

[6]: indicated significance test refers to the overall test of the interaction species:DGW

Table S6 Results of the second reduced linear mixed model (LMM) for the upper canopy. Indicated are estimates, standard errors (SE), significance tests, confidence limits and the range of estimates obtained when dropping levels of grouping factors one at a time. Ace_pse= *Acer pseudoplatanus*, Car_bet= *Carpinus betulus*, Que_rob= *Quercus robur*, Til_cor= *Tilia cordata*; spec= species; ADB= ash dieback, as effective basal area change of ash trees; DGW = distance to ground water; density= plot density; Δdamage= delta damage degree; ΔT= time interval between first and second inventory.

Term	Estimate	SE	F	Num df	Den df	p	lower CI	upper CI	min	max
Intercept	-3.450	0.091	[1]	[1]	[1]	[1]	-3.638	-3.266	-3.494	-3.410
spec.Ace_pse[2,5]	-0.237	0.094	13.095	4	31.781	**<0.001**	-0.434	-0.053	-0.281	-0.186
spec.Car_bet[2]	-0.339	0.124	[1]	[1]	[1]	[1]	-0.600	-0.088	-0.416	-0.299
spec.Que_rob[2]	-0.481	0.088	[1]	[1]	[1]	[1]	-0.669	-0.319	-0.534	-0.439
spec.Til_cor[2]	-0.516	0.094	[1]	[1]	[1]	[1]	-0.716	-0.334	-0.571	-0.482
ADB[3]	-0.032	0.029	[1]	[1]	[1]	[1]	-0.093	0.031	-0.042	-0.025
DGW[3]	-0.008	0.030	[1]	[1]	[1]	[1]	-0.066	0.054	-0.029	0.014
density[3]	-0.086	0.031	7.727	1	33.071	**0.009**	-0.150	-0.017	-0.102	-0.067
Δdamage[3]	-0.071	0.018	15.547	1	61.825	**<0.001**	-0.103	-0.034	-0.080	-0.064
vitality.normal[4]	0.179	0.060	4.526	2	32.916	**0.018**	0.073	0.294	0.151	0.205
vitality.vital[4]	0.251	0.135	[1]	[1]	[1]	[1]	-0.023	0.532	0.185	0.304
ΔT[3]	-0.004	0.029	0.022	1	42.091	0.882	-0.065	0.057	-0.019	0.017
ADB:DGW	-0.091	0.028	10.497	1	37.660	**0.003**	-0.151	-0.036	-0.105	-0.078

[1]: not shown because of being of very limited interpretability since term is involved in a higher order interaction
[2]: species was dummy coded with Ace_pla being the reference level
[3]: z-transformed to mean = 0 and sd = 1; mean and sd of the original variables are presented in table S5
[4]: vitality was dummy coded with suffering being the reference level
[5]: indicated significance test refers to the overall test of species

Table S7 Results of the linear mixed model (LMM) for the lower canopy. Indicated are estimates, standard errors (SE), significance tests, confidence limits and the range of estimates obtained when dropping levels of grouping factors one at a time. Ace_pla= *Acer platanoides*, Ace_pse= *Acer pseudoplatanus*, Car_bet= *Carpinus betulus*, Til_cor= *Tilia cordata*, Ulm_spe= *Ulmus spec.*; spec= species; ADB= ash dieback, as effective basal area change of ash trees; DGW = distance to ground water; density= plot density; Δdamage= delta damage degree; ΔT= time interval between first and second inventory.

Term	Estimate	SE	F	Num df	Den df	p	lower CI	upper CI	min	max
Intercept	-2.835	0.076	(1)	(1)	(1)	(1)	-2.990	-2.687	-2.885	-2.815
specAce_pla(2)	0.281	0.100	(1)	(1)	(1)	(1)	0.098	0.482	0.252	0.334
spec.Ace_pse(2)	-0.116	0.075	(1)	(1)	(1)	(1)	-0.256	0.034	-0.132	-0.071
spec.Car_bet(2)	-0.392	0.092	(1)	(1)	(1)	(1)	-0.577	-0.223	-0.420	-0.347
spec.Til_cor(2)	-0.463	0.071	(1)	(1)	(1)	(1)	-0.604	-0.319	-0.484	-0.429
spec.Ulm_spe(2)	-0.066	0.077	(1)	(1)	(1)	(1)	-0.214	0.086	-0.088	-0.028
ADB(3)	-0.056	0.069	(1)	(1)	(1)	(1)	-0.191	0.080	-0.083	-0.016
DGW(3)	-0.110	0.061	(1)	(1)	(1)	(1)	-0.231	0.015	-0.181	-0.078
density(3)	-0.078	0.029	7.347	1	55.598	**0.009**	-0.142	-0.013	-0.090	-0.052
Δdamage(3)	-0.083	0.016	28.545	1	55.291	**<0.001**	-0.116	-0.051	-0.087	-0.078
vitality.normal(4)	0.331	0.033	50.593	2	67.378	**<0.001**	0.264	0.396	0.322	0.341
vitality.vital(4)	0.372	0.211	(1)	(1)	(1)	(1)	-0.151	0.806	0.261	0.431
ΔT(3)	-0.069	0.031	5.100	1	56.340	**0.028**	-0.130	0.000	-0.084	-0.053
specAce_pla:ADB(6)	0.095	0.104	(1)	(1)	(1)	(1)	-0.109	0.298	0.056	0.182
spec.Ace_pse:ADB	0.050	0.065	(1)	(1)	(1)	(1)	-0.086	0.170	0.015	0.074
spec.Car_bet:ADB	-0.075	0.083	(1)	(1)	(1)	(1)	-0.240	0.083	-0.108	-0.018
spec.Til_cor:ADB	-0.161	0.070	(1)	(1)	(1)	(1)	-0.308	-0.015	-0.186	-0.123
spec.Ulm_spe:ADB	0.090	0.071	(1)	(1)	(1)	(1)	-0.057	0.226	0.057	0.113
specAce_pla:DGW(7)	0.148	0.089	(1)	(1)	(1)	(1)	-0.015	0.326	0.094	0.223
spec.Ace_pse:DGW	0.036	0.063	(1)	(1)	(1)	(1)	-0.088	0.162	0.010	0.109
spec.Car_bet:DGW	-0.104	0.079	(1)	(1)	(1)	(1)	-0.278	0.048	-0.129	-0.029
spec.Til_cor:DGW	0.013	0.068	(1)	(1)	(1)	(1)	-0.125	0.150	-0.008	0.079
spec.Ulm_spe:DGW	0.098	0.064	(1)	(1)	(1)	(1)	-0.026	0.230	0.081	0.168
ADB:DGW	-0.073	0.055	(1)	(1)	(1)	(1)	-0.181	0.050	-0.108	0.012
specAce_pla:ADB:DGW(5)	-0.056	0.094	3.327	5	108.750	**0.008**	-0.269	0.128	-0.153	-0.016

spec.Ace_pse:ADB:DGW	-0.045	0.054	(1)	(1)	(1)	(1)	-0.157	0.055	-0.117	-0.017
spec.Car_bet:ADB:DGW	-0.197	0.093	(1)	(1)	(1)	(1)	-0.394	-0.011	-0.286	-0.153
spec.Til_cor:ADB:DGW	0.153	0.075	(1)	(1)	(1)	(1)	-0.003	0.291	0.063	0.175
spec.Ulm_spe:ADB:DGW	0.041	0.055	(1)	(1)	(1)	(1)	-0.082	0.146	-0.032	0.065

(1): not shown because of being of very limited interpretability since term is involved in a higher order interaction
(2): species was dummy coded with Ace_cam being the reference level
(3): z-transformed to mean = 0 and sd = 1; mean and sd of the original variables are presented in table S5
(4): vitality was dummy coded with suffering being the reference level
(5): indicated significance test refers to the overall test of the 3-way interaction species:ADB:DGW

Table S8 Mean and standard deviations (SD) of the original predictor variables for the upper and lower canopy model.

	upper canopy		lower canopy	
predictor	mean	SD	mean	SD
ADB	0.340	0.360	0.511	0.428
DGW	2.030	0.517	1.820	0.500
density	7.693	1.504	7.787	1.367
Δdamage	2.114	1.687	1.803	1.863
ΔT	5.717	1.485	5.705	1.486

Table S9 Mean slopes calculated from 1000 parametric bootstraps per species, moist and dry sites (i.e. 25% and 75% percentile for distance to groundwater, DGW), for the upper canopy and lower canopy. We used bootstrapped fitted values to conduct post-hoc pairwise comparisons, comparing growth responses to ash dieback (ADB) between moist and dry sites for each species as well as between all species for moist and dry sites, respectively, separately for the lower and upper canopy.

	upper canopy		lower canopy	
species	moist sites	dry sites	moist sites	dry sites
A. campestre	NA	NA	-0.004	-0.106
A. platanoides	0.165	-0.052	0.140	-0.049
A. pseudoplatanus	-0.013	-0.118	0.080	-0.089
C. betulus	0.109	-0.113	0.074	-0.324
T. cordata	0.032	-0.057	-0.279	-0.164
Q. robur	0.044	-0.123	NA	NA
U. spec.	NA	NA	0.059	0.009

Table S10 Results of the post-hoc pairwise comparisons of growth responses to ash dieback between moist and dry sites (i.e. 25% and 75% percentile for distance to groundwater), respectively, for the upper and lower canopy. Shown are the p-values. Significant results are indicated in bold.

species	upper canopy	lower canopy
A. campestre	NA	**0.034**
A. platanoides	0.408	0.518
A. pseudoplatanus	0.270	**0.006**
C. betulus	0.374	**<0.001**
T. cordata	0.232	0.874
Q. robur	0.114	NA
U. spec.	NA	0.442

Table S11 Results of the post-hoc pairwise comparisons of growth responses to ash dieback between all species on moist and dry sites (i.e. 25% and 75% percentile for distance to groundwater), respectively, for the upper canopy. Shown are the p-values.

moist sites					
species	*A. platanoides*	*A. pseudoplatanus*	*C. betulus*	*Q. robur*	*T. cordata*
A. platanoides	NA	0.584	0.940	0.862	0.938
A. pseudoplatanus	0.584	NA	0.400	0.146	0.406
C. betulus	0.940	0.400	NA	0.886	0.866
Q. robur	0.862	0.146	0.886	NA	0.712
T. cordata	0.938	0.406	0.866	0.712	NA
dry sites					
species	*A. platanoides*	*A. pseudoplatanus*	*C. betulus*	*Q. robur*	*T. cordata*
A. platanoides	NA	0.522	0.776	0.592	0.504
A. pseudoplatanus	0.522	NA	0.816	0.618	0.918
C. betulus	0.776	0.816	NA	0.906	0.742
Q. robur	0.592	0.618	0.906	NA	0.624
T. cordata	0.504	0.918	0.742	0.624	NA

Table S12 Results of the post-hoc pairwise comparisons of growth responses to ash dieback between all species on moist and dry sites (i.e. 25% and 75% percentile for distance to groundwater), respectively, for the lower canopy. Shown are the p-values. Significant results are indicated in bold.

moist sites						
species	*A. campestre*	*A. platanoides*	*A. pseudoplatanus*	*C. betulus*	*T. cordata*	*U. spec.*
A. campestre	NA	0.244	0.226	0.450	**0.004**	0.418
A. platanoides	0.244	NA	0.624	0.588	**<0.001**	0.462
A. pseudoplatanus	0.226	0.624	NA	0.914	**<0.001**	0.744
C. betulus	0.450	0.588	0.914	NA	**<0.001**	0.854
T. cordata	0.004	<0.001	<0.001	<0.001	NA	<0.001
U. spec.	0.418	0.462	0.744	0.854	**<0.001**	NA
dry sites						
species	*A. campestre*	*A. platanoides*	*A. pseudoplatanus*	*C. betulus*	*T. cordata*	*U. spec.*
A. campestre	NA	0.674	0.864	**0.066**	0.488	0.184
A. platanoides	0.674	NA	0.720	**0.022**	0.338	0.566
A. pseudoplatanus	0.864	0.720	NA	**0.018**	0.306	0.194
C. betulus	0.066	0.022	0.018	NA	0.152	<0.001
T. cordata	0.488	0.338	0.306	0.152	NA	0.026
U. spec.	0.184	0.566	0.194	**<0.001**	**0.026**	NA

Table S13 Overview of important ecological attributes influencing the growth response of the main tree species in the Leipzig floodplain forest, assembled according to Röhrig and Dengler, 1980; Burschel and Huss, 1987; Ellenberg and Leuschner, 2010; Matyssek *et al.*, 2010; Schütt, Schnuck and Stimm, 2011; Jäger, 2017; Leuschner and Meier, 2018; Schwedt, 2021. Adapted from Dontschev (2023).

Species	max. height (m)	max. DBH (cm)	max. age (years)	Drought sensitivity (adult)[1]	Indicator value humidity[2]	Flood Tolerance[3]	Indicator value light[2]	Shade tolerance[4]
A. campestre	22	100	100-200	2	F5	3	L 5	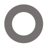
A. platanoides	35	100	150-300	3	Fx	2	L 4	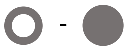
A. pseudoplatanus	35	200	300-500	3	F6	1	L 4	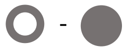
C. betulus	25	120	150-250	3	Fx	2	L 4	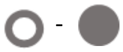
F. excelsior	35-40	200	200-300	4	Fx	3	L 4	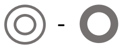
Q. robur	20-50	300	500-900	1	Fx	3	L 7	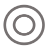
T. cordata	20-35	300	400-1000	2	F5	2	L 5	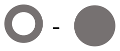
U. glabra	40-48	150	400	3	F6	2	L 4	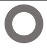
U. leavis	25-35	200	250-400	4	F8=	4*	L 4	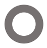
U. minor	35	100	300-400	2	Fx~	3	L 5	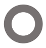

[1] **Drought sensitivity** (according to Leuschner & Meier (2018))**:**
1 = very low, 2 = low, 3 = medium, 4 = high, 5 = very high
[2] **Description of Ellenberg's ecological indicator values** (Jäger, 2017)
Humidity:
F5 - fresh site indicator, mostly on moderately moist soils, absent on wet soils and on soils that dry out frequently
F6 - standing between 5 and 7
F7 - moisture indicator, predominantly on well-damp but not wet soils
F8 - standing between 7 and 9
F9 - wetness indicator, heavyweight often on very wet soils (poor air conditions)
~ Indicator for strong alternation
= Flood indicator, on more or less regularly flooded soils
x Indifferent behaviour, i.e., wide amplitude or uneven behaviour in different areas.
Light:
L3 - shade plant, mostly at < 5% relative brightness, but also in brighter places
L 4 - standing between 3 and 5
L 5 - semi-shade plant, only exceptionally in full light, but mostly at > 10 % relative illuminance at the time of full foliage of deciduous plants with diffuse lighting.
L 7 - half-light plant, mostly in full light, but also in shade up to about 30% relative illuminance at the time of full foliage of deciduous plants with diffuse lighting.

[3]**Shade tolerance:**

shade intolerant: ◎ intermediate shade tolerant: ○ shade tolerant: ●

References

Burschel, P., & Huss, J. (1987). *Grundriß des Waldbaus – Ein Leitfaden für Studium und Praxis*. Verlag Paul Parey, Hamburg und Berlin.

Dontschev, M. (2023). *Tree growth responses to canopy disturbance in dependence of tree species, stand structure and water availability in a Central-European floodplain forest* [Bachelorarbeit]. Technische Universität Berlin.

Ellenberg, H., & Leuschner, C. (2010). *Vegetation Mitteleuropas und der Alpen in ökologischer, dynamischer und historischer Sicht* (6., erweiterte Auflage).

Jäger, E. J. (Ed.). (2017). *Rothmaler - Exkursionsflora von Deutschland. Gefäßpflanzen: Grundband*. Springer Berlin Heidelberg. https://doi.org/10.1007/978-3-662-49708-1

Lenz, H., Straßer, L., Baumann, M., & Baier, U. (2012). Boniturschlüssel zur Einstufung der Vitalität von Alteschen. *AFZ-DerWald*, *3/2012*, 18–19.

Leuschner, C., & Meier, I. C. (2018). The ecology of Central European tree species: Trait spectra, functional trade-offs, and ecological classification of adult trees. *Perspectives in Plant Ecology, Evolution and Systematics*, *33*, 89–103. https://doi.org/10.1016/j.ppees.2018.05.003

Matyssek, R., Fromm, J., Rennenberg, H., & Roloff, A. (2010). *Biologie der Bäume von der Zelle zur globalen Ebene* (191 Abbildungen, 32 Tabellen, UTB 8450). Verlag Eugen Ulmer Stuttgart, Wollgrasweg 41, 70599 Stuttgart (Hohenheim).

Röhrig, E., & Dengler, A. (1980). *Waldbau auf ökologischer Grundlage – Der Wald als Vegetationstyp und seine Bedeutung für den Menschen*. Parey P. Hamburg und Berlin.

Schütt, P., Schnuck, H.-J., & Stimm, B. (2011). *Lexikon der Baum- und Straucharten—Das Standardwerk der Forstbotanik—Morphologie, Pathologie, Ökologie und Systematik*

wichtiger Baum- und Straucharten. Nikol Verlagsgesellschaft mbH & Co. KG, Hamburg, 2011, by WILEY-VCH Verlag GmnH & Co. KGaA,.

Schwedt, G. (2021). *Forstbotanik—Vom Baum zum Holz*. Springer Spektrum. https://doi.org/10.1007/978-3-662-63407-3
